# Identifying dysregulated immune cell subsets following volumetric muscle loss with pseudo-time trajectories

**DOI:** 10.1038/s42003-023-04790-6

**Published:** 2023-07-19

**Authors:** Lauren A. Hymel, Shannon E. Anderson, Thomas C. Turner, William Y. York, Hongmanlin Zhang, Adrian R. Liversage, Hong Seo Lim, Peng Qiu, Luke J. Mortensen, Young C. Jang, Nick J. Willett, Edward A. Botchwey

**Affiliations:** 1 Department of Biomedical Engineering, Georgia Institute of Technology, Atlanta, GA USA; 2 Petit Institute for Bioengineering and Bioscience, Georgia Institute of Technology, Atlanta, GA USA; 3 School of Materials Science and Engineering, Georgia Institute of Technology, Atlanta, GA USA; 4 School of Chemical, Materials, and Biomedical Engineering, University of Georgia, Athens, GA USA; 5 Regenerative Bioscience Center, Rhodes Center for ADS, University of Georgia, Athens, GA USA; 6 Department of Orthopaedics, Emory University, Atlanta, GA USA; 7 Atlanta Veterans Affairs Medical Center, Decatur, GA USA; 8 Phil and Penny Knight Campus for Accelerating Scientific Impact, University of Oregon, Eugene, OR USA; 9 The Veterans Affairs Portland Health Care System, Portland, OR USA

**Keywords:** Chronic inflammation, Trauma

## Abstract

Volumetric muscle loss (VML) results in permanent functional deficits and remains a substantial regenerative medicine challenge. A coordinated immune response is crucial for timely myofiber regeneration, however the immune response following VML has yet to be fully characterized. Here, we leveraged dimensionality reduction and pseudo-time analysis techniques to elucidate the cellular players underlying a functional or pathological outcome as a result of subcritical injury or critical VML in the murine quadriceps, respectively. We found that critical VML resulted in a sustained presence of M2-like and CD206^hi^Ly6C^hi^ ‘hybrid’ macrophages whereas subcritical defects resolved these populations. Notably, the retained M2-like macrophages from critical VML injuries presented with aberrant cytokine production which may contribute to fibrogenesis, as indicated by their co-localization with fibroadipogenic progenitors (FAPs) in areas of collagen deposition within the defect. Furthermore, several T cell subpopulations were significantly elevated in critical VML compared to subcritical injuries. These results demonstrate a dysregulated immune response in critical VML that is unable to fully resolve the chronic inflammatory state and transition to a pro-regenerative microenvironment within the first week after injury. These data provide important insights into potential therapeutic strategies which could reduce the immune cell burden and pro-fibrotic signaling characteristic of VML.

## Abstract (web summary)

The immune cell dynamics that influence the pathological phenotype of a critical volumetric muscle loss (VML) injury that cannot regenerate versus a subcritical injury capable of regeneration are characterized using quadriceps VML mouse models.

## Introduction

Following acute muscle injury, skeletal muscle’s robust regenerative response relies on the prompt and proper coordination of immune cells. The cellular dynamics of myeloid and lymphoid cell trafficking into and out of the muscle coincide with each stage of the muscle regenerative process^1^. Following muscle injury, there is degeneration and necrosis of damaged myofibers^2^, triggering the invasion of neutrophils that peak within hours following injury and drop off after 24 h^3^. As neutrophils secrete tumor necrosis factor (TNF) and interferon-γ (IFN-γ), monocyte derived pro-inflammatory phagocytic (M1) macrophages infiltrate the muscle to aid in the removal of tissue debris and propagate pro-inflammatory signals by secretion of cytokines^1^. Both TNF-α and IFN-γ play a role in macrophage induction and skeletal muscle regeneration by silencing Pax7 and preventing the expression of MyoD to maintain a required pool of skeletal muscle stem/satellite cells (MuSCs), in an activated, proliferative state^1,4^. M1 macrophage secretion of TNF-α is also a mechanism to induce fibroadipogenic progenitor cell (FAP) clearance by apoptosis^5^. FAPs are muscle- resident mesenchymal stromal cells, and their regulated apoptosis induced by TNF-α is a key signaling event for healthy extracellular matrix (ECM) secretion^6^.

Once in tissue, classical Ly6C^hi^ monocytes can convert into non-classical Ly6C^lo^ monocytes which are biased progenitors of pro-regenerative (M2) macrophages, as we have shown previously^7^. The transition to an anti-inflammatory, interleukin (IL)-10 and transforming growth factor (TGF)-β rich environment corresponds with both a transition to M2 macrophages around 4–7 days post injury^8,9^ as well as the differentiation and growth stages of myogenesis^10^. As the skeletal muscle regenerates, myeloid cells traffic out of the tissue by 2 weeks post-injury^11^. This coordinated response of myeloid cells is crucial for the proper regeneration of skeletal muscle, as the depletion or altered polarization of macrophages has been shown to increase adipose and fibrotic tissue deposition while reducing regenerated myofiber cross-sectional area^12,13^.

In addition to myeloid derived cells, lymphoid-derived T-cells also respond to muscle injury. Peak infiltration of CD4^+^ helper and CD8^+^ cytotoxic T cells occurs at 3 days post-injury, returning to baseline levels gradually by day 14^14^. Regulatory T cells (T_reg_ cells) infiltrate the muscle after acute injury with similar kinetics to that of M2 macrophages—peaking at day 4 post-injury^15^. The T cell response has been implicated in the maintenance of myeloid cell infiltration. The depletion of CD8^+^ T cells have been shown to reduce skeletal muscle regeneration through a reduction in the recruitment of pro-inflammatory M1 macrophages^14^. Similarly, depletion of T_reg_ cells impairs muscle repair and prolongs inflammation^15^, which could be attributed to the need of T_reg_ cells for the transition from M1 to M2 macrophage phenotype, the ability of T_reg_ cells to limit IFN-γ and macrophage accrual, or the need for T_reg_ derived IL-10^15–17^. The regulation of myeloid and lymphoid immune cell infiltration and clearance works in concert with the stages of myogenesis for prompt muscle regeneration after acute injury.

By contrast, following large volume skeletal muscle loss that exceeds the regenerative threshold (volumetric muscle loss, VML), chronic inflammation produces an inhospitable microenvironment that does not allow for muscle regeneration. Instead, healthy muscle fibers are replaced by non-contractile fibrotic tissue resulting in chronic loss of function and permanent disability^18–20^. This inflammatory microenvironment after VML has been previously characterized as having an enduring presence of CD4^+^ and CD8^+^ T cells, as well as improper macrophage polarization^6,11,15,21,22^. Moreover, a muscle environment that is rich with both pro- and anti- inflammatory factors (i.e. TNF-α and TGF-β) can cause sustained presence of M2 macrophages that shift from ‘pro-reparative’ to ‘pro-fibrotic’ and drive FAPs to a pathological state rather than a regenerative one^23^. However, the initial immune cell response following a critical muscle defect that often leads to chronic inflammation and fibrotic outcomes has not been elucidated. Previously, we characterized multiple full-thickness biopsy punch injuries in the quadriceps muscles as a VML model in C57BL/6 mice. We found that a 2 mm diameter biopsy punch injury caused damage to the tissue, but the muscle was able to regenerate without significant fibrotic scarring (denoted as subcritical injury). However, a 3 mm injury caused persistent fibrotic scarring and inflammation through 4 weeks following injury (denoted as critical VML injury)^24^.

**Fig. 1:**
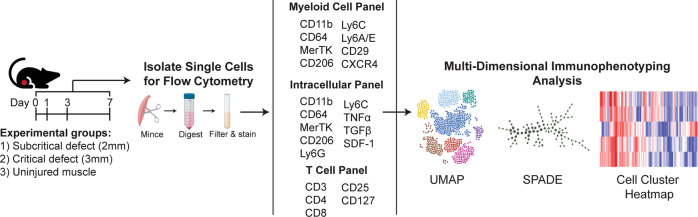
Graphical workflow of multiparameter pseudo-time analysis applied to single cell flow cytometry data. Animals received either a full-thickness, unilateral subcritical injury (2 mm diameter biopsy) or critical VML injury (3 mm diameter biopsy) to the left quadriceps. Uninjured left quadriceps muscle (indicated as day 0 in timeline) from naïve mice was used as a control. At 1, 3, or 7 days post injury, tissue was excised, digested, and single cells were isolated and stained for flow cytometric analysis. Dimensionality reduction and pseudo-time clustering algorithms, namely UMAP and SPADE, were employed to characterize the temporal immune response of myeloid and lymphoid cells following VML. Created with BioRender.com.

A population of cells captured at the same time via high-dimensional immune cell profiling includes many distinct intermediate differentiation states of cells; however, classical analytical techniques only evaluate their average properties and thus mask trends occurring across individual cells and subpopulations^25–27^. In this study, we approached this challenge by employing a unique dimensionality reduction and clustering analytical strategy that combines both uniform manifold approximation and projection (UMAP) and spanning-tree progression analysis of density-normalized events (SPADE); this analytical approach captures the functional heterogeneity of the early immune cell response and dynamics after an injury event and was applied to the scenario of critical VML injury (Fig. 1). UMAP was utilized for graphing single cells isolated from multiparameter flow cytometric analysis into a lower dimensional representation^28^. Furthermore, we leveraged SPADE clustering to reconstruct cellular hierarchies and transitional states that can be inferred through time—a concept known as ‘pseudo-time’^25,29^. By generating SPADE dendrograms with pooled sample data across timepoints, single cells were clustered into distinct nodes and then ordered along a trajectory to temporally track cellular lineages and progressions. These dimensionality reduction and clustering tools provide greater insights into the underlying heterogeneous cell populations contributing to the early immune response after VML injury.

Previous studies have comprehensively reconstructed the temporal response of cell populations following more established, acute models of muscle injury^30,31^. The objective of this study was to characterize the immune cell dynamics that influence the pathological phenotype of a traumatic VML injury that cannot endogenously regenerate versus a full-thickness injury still capable of regeneration (2 mm subcritical injury) using our quadriceps VML model in C57BL/6 mice. We hypothesized that critical VML would lead to persistent elevation of key immune cell subpopulations, particularly those which are associated with promoting fibrosis.

## Results

### UMAP visualization of immune cell recruitment to critical VML injury reveals persistent myeloid cell response

The flow panel designed for myeloid cell characterization contained antibodies for CD11b, CD64, and MerTK for identifying all myeloid cells, including parent monocyte and macrophage populations. CD11b, a cell surface leukocyte integrin expressed on myeloid-lineage cells, served as our canonical myeloid cell marker^32^. CD64, a high-affinity Fc receptor, and MerTK, a receptor tyrosine kinase and mediator of phagocytosis, are commonly used to delineate tissue monocytes (CD11b^+^CD64^+^MerTK^-^SSC^lo^) from macrophages (CD11b^+^CD64^+^MerTK^+^)^33^. Ly6C antigen further differentiates between classical Ly6C^hi^ and non-classical Ly6C^lo^ murine monocytes^7,32^, and together with mannose receptor CD206, can be used to classify pro- inflammatory M1-like macrophages (Ly6C^hi^CD206^lo^) from alternatively activated M2-like macrophages (Ly6C^lo^CD206^hi^)^34^.

**Fig. 2:**
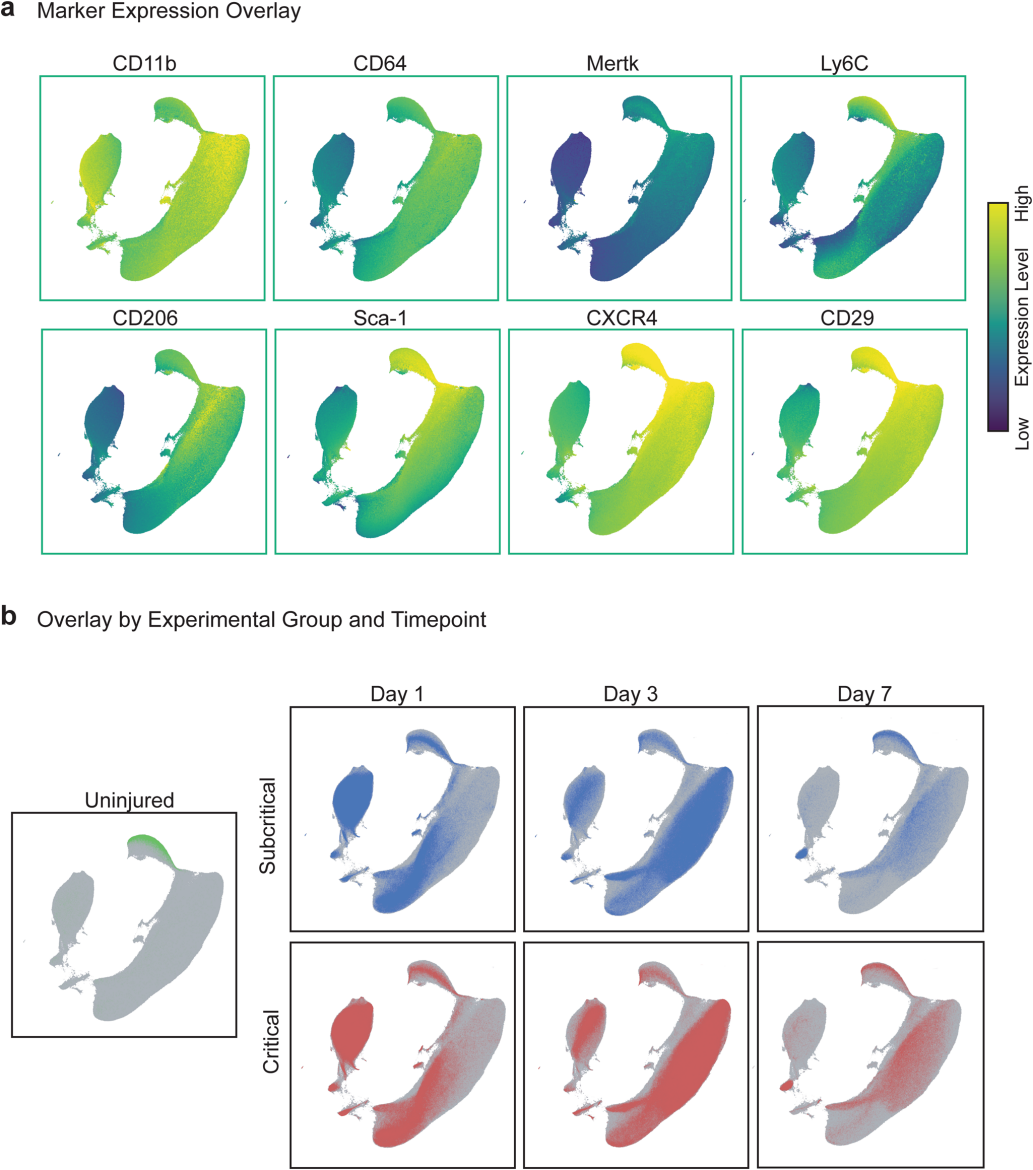
Persistent myeloid cell response in critical VML injury as visualized by UMAP analysis. UMAP representation comprised of single cell flow cytometry data from explanted quadriceps muscle at days 1, 3, and 7 post injury in addition to uninjured quadriceps. **a** UMAP projection overlaid with marker expression values of each targeted surface protein. Marker expression levels range from dark blue to yellow, representing low to high expression, respectively. **b** CD11b^+^ cells from uninjured quadriceps overlaid onto UMAP (green). CD11b^+^ cells from quadriceps that received a subcritical injury (blue, top) and those from quadriceps that received a critically sized VML injury (red, bottom) overlaid onto the UMAP at days 1, 3, and 7 post injury (left to right). UMAP constructed from *n* = 4 biologically independent animals per experimental group.

Following subcritical injury (2 mm) or critical (3 mm) VML injury, immune cells were isolated from injured quadriceps muscles and analyzed at 1, 3, and 7 days post injury via biplot gating and multi-dimensional immunophenotyping analysis of flow cytometry data (Fig. 1, bi-plot gating in Supplementary Fig. 1). Quadriceps explanted from naïve (uninjured) mice were used as a control. All CD11b^+^ myeloid cells from uninjured and injured animals at all timepoints (days 1, 3, and 7) were used to construct a UMAP plot that graphs single cells by their surface marker profiles, where further distances between cells indicates dissimilar cellular phenotypes (Fig. 2). Surface marker expression values are represented on the UMAP, ranging from dark blue (low expression) to yellow (high expression) (Fig. 2a). Each CD11b^+^ cellular event is represented as a dot on the UMAP, with those coming from uninjured quadriceps overlaid in green and those from subcritical injury or critical VML injured quadriceps overlaid in blue and red, respectively (Fig. 2b).

At day 1, myeloid cells from injured quadriceps qualitatively localized near the bottom left of the UMAP plot, indicating that day 1 myeloid cells have low expression of CD206, MerTK, and CXCR4, chemokine receptor for stromal cell- derived factor 1 (SDF-1), but highly express Ly6C (Fig. 2a, b). In contrast, the upper right portion of the UMAP is enriched for day 3 and day 7 cells, representing a phenotypic switch to increased CD206, CD64, MerTK, stem cell antigen-1 (Sca-1), CD29 (integrin β1), and CXCR4 expression and a lower expression of Ly6C by day 7. While the overlay of CD11b^+^ events is located similarly in UMAP space between subcritical injury and critical VML, the number of events is remarkably different. At days 3 and 7, critical VML injuries presented with a higher number of myeloid cells within the UMAP compared to subcritical injuries, suggesting that inflammation was not completely resolved by day 7.

**Fig. 3:**
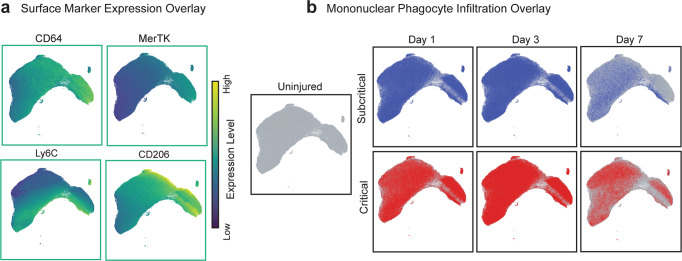
UMAP analysis reveals accumulation of mononuclear phagocytes in critically sized VML injuries. UMAP generated from flow cytometry gated CD11b^+^CD64^+^MerTK^+^ macrophages and CD11b^+^CD64^+^MerTK^-^SSC^lo^ monocytes extracted from quadriceps tissue (uninjured and at days 1, 3, and 7 post injury). **a** UMAP projection overlaid with surface maker expression values. Marker expression levels range from dark blue to yellow, representing low to high expression, respectively. **b** Visualization of mononuclear phagocyte infiltration in quadriceps that received no injury (uninjured), subcritical injury (blue, top) or critically sized injury (red, bottom) at each timepoint post injury (days 1, 3, and 7). UMAP constructed from *n* = 4 biologically independent animals per experimental group.

The increased expression of CD64 and MerTK in the upper right portion of the UMAP suggests that mononuclear phagocyte populations are present at days 3 and 7 post injury. Thus, a UMAP plot comprised of all CD11b^+^CD64^+^MerTK^-^SSC^lo^ monocytes and CD11b^+^CD64^+^MerTK^+^ macrophages pooled from all samples across all three timepoints was constructed (Fig. 3). Expression levels of surface markers characterizing monocyte and macrophage subsets were overlaid onto UMAP plots to illustrate the expression profile of unique mononuclear phagocyte subpopulations (Fig. 3a). While the location of overlaid monocytes and macrophages appear similar between subcritical injury and critical VML at days 1 and 3, the top middle and far right sections of the UMAP largely contain day 7 cells from critical VML (Fig. 3b). Notably, these day 7 critical VML mononuclear phagocytes are positioned in UMAP space based on their low expression of Ly6C and high expression of CD206 (Fig. 3a, b). This retention of mononuclear phagocytes may be explained by the increased secretions of pro-inflammatory cytokines within the injury niche as indicated by an upwards trend in TNF-α expressing neutrophils at day 7 in critical VML (Supplementary Fig. 2).

### Monocyte subpopulations facilitate the chronic inflammation characteristic of critical VML

**Fig. 4:**
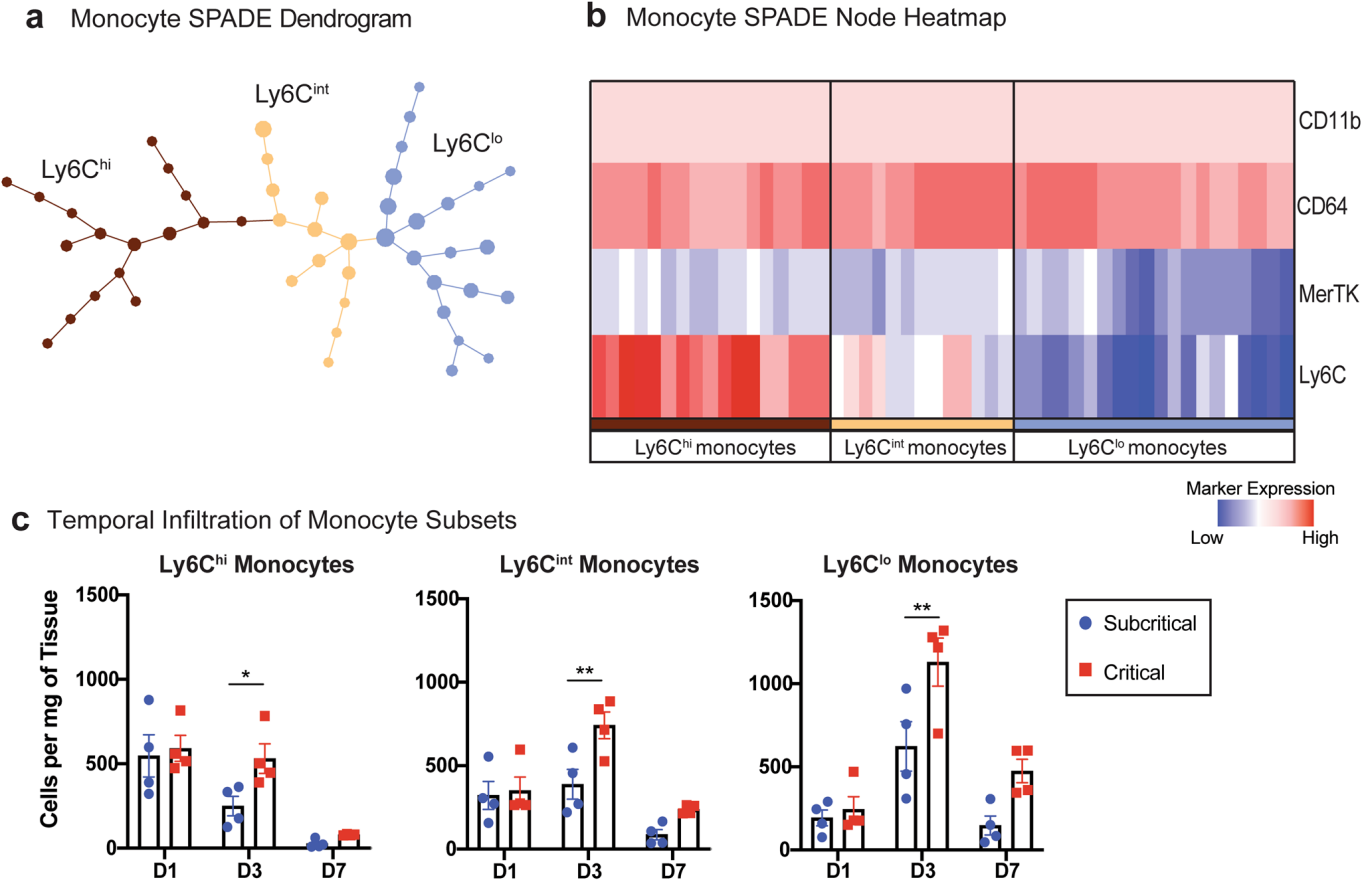
Unbiased SPADE clustering identifies distinct monocyte subpopulations that are significantly increased in critically sized injuries 3 days post VML. SPADE dendrogram generated from all CD11b^+^CD64^+^MerTK^-^SSC^lo^ monocytes isolated from quadriceps muscle of mice which were uninjured or those which received a subcritical injury or critical VML injury (at days 1, 3, and 7 post injury). **a** Monocyte SPADE dendrogram annotated by nodes containing monocytes with low (Ly6C^lo^), intermediate (Ly6C^int^), or high (Ly6C^hi^) expression of Ly6C. **b** Protein signature of each SPADE node represented as a heatmap. SPADE nodes classified according to their expression of Ly6C. Color annotation of SPADE nodes on the dendrogram align with those on the heatmap. **c** Quantification of monocyte subsets (per mg of tissue) within quadriceps muscle at each timepoint post injury. Data presented as mean ± S.E.M. Statistical analyses performed include two-way ANOVA with *Sidak* multiple comparisons between injury sizes at each timepoint. *p < 0.05, **p < 0.01. *n* = 4 biologically independent animals per experimental group. D1: day 1, D3: day 3, D7: day 7.

To overcome the intrinsic challenge of subjective cell subset identification presented by traditional methods for analyzing multiparameter single-cell data, unsupervised SPADE clustering analysis was applied to our flow cytometry data. SPADE groups the cells into distinct clusters, or nodes, by similar marker expression and then arranges these nodes into branching trajectories that infer cellular transitions through time^35^. We constructed a SPADE dendrogram with CD11b^+^CD64^+^MerTK^-^SSC^lo^ monocytes from all samples and all timepoints (days 1, 3, and 7) after injury, including uninjured controls. The SPADE dendrogram is color annotated into three monocyte subpopulations identified by the median Ly6C expression of each node (Supplementary Fig. 3a). A corresponding SPADE node heatmap, where each column correlates to one SPADE node and rows correlate to surface marker expression, indicates the distinction of these three unique monocyte subsets based on Ly6C expression (Fig. 4a, b). From left to right, the phenotype of the monocyte SPADE nodes transition from Ly6C^hi^ to Ly6C^lo^, illustrating the dynamic biological progression of monocytes after VML.

The concentration of cells within each of the three SPADE-identified monocyte subpopulations was quantified for all timepoints (Fig. 4c). For all subsets, there was a significant increase in the number of monocytes present in critical VML at day 3 post injury. Ly6C^int^ monocytes—which would typically be left out of traditional gating strategies between the ‘high’ and ‘low’ expressing gates—and Ly6C^lo^ monocytes both reached peak concentrations at day 3 with Ly6C^lo^ monocytes reaching the highest concentration of all the subpopulations at any timepoint. To evaluate the cytokine expression profile of these monocyte subsets, we again performed flow cytometry analysis from explanted quadriceps at days 3 and 7 after subcritical injury or critical VML. There were no differences found in the concentration of TNF-α^+^ or SDF-1^+^ monocyte subsets infiltrating subcritical injury and critical VML at day 3 (Supplementary Fig. 3b–d). However, at day 7, the number of Ly6C^int^ monocytes expressing TNF-α and Ly6C^hi^ monocytes expressing SDF-1 were significantly elevated in critical VML. Almost no monocytes, regardless of subset classification, expressed TNF-α or SDF-1 in subcritical injuries at day 7 (Supplementary Fig. 3c, d).

### Aberrant macrophage subsets dysregulated following critical VML injury may contribute to fibrotic VML pathology

**Fig. 5:**
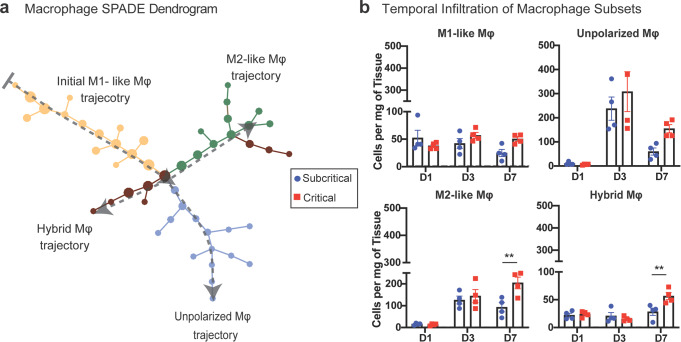
SPADE identification of macrophage subsets uncovers two unique populations expressing CD206 which are significantly increased in critical VML injuries at day 7. SPADE dendrogram generated from all CD11b^+^CD64^+^MerTK^+^ macrophages isolated from quadriceps muscle of mice which were uninjured or those which received a subcritical injury or critically sized VML injury (at days 1, 3, and 7 post injury). **a** Macrophage SPADE dendrogram annotated based on CD206 and Ly6C expression to designate nodes to a M1-like, M2-like, unpolarized, or hybrid macrophage phenotype. Gray arrow indicates general movement over time following injury. **b** Quantification of macrophage subsets (per mg of tissue) within quadriceps muscle at each timepoint post injury. Dashed gray line represents average value for uninjured controls. Data presented as mean ± S.E.M. Statistical analyses performed include two-way ANOVA with *Sidak* test for multiple comparisons between injury sizes at each timepoint. **p < 0.01. *n* = 4 biologically independent animals per experimental group. D1: day 1, D3: day 3, D7: day 7, Mφ: macrophage.

All CD11b^+^CD64^+^MerTK^+^ macrophages, from uninjured and injured quadriceps (at days 1, 3, and 7 post injury) were used to generate a SPADE dendrogram (Fig. 5a). SPADE clustered the macrophages into nodes ordered along 4 marked trajectories, each color-coded, characterized by the surface marker expression of each node (Supplementary Fig. 4a, b). Most macrophages present within the injured muscle at day 1 are clustered within nodes of the initial M1-like trajectory, characterized by its Ly6C^hi^CD206^lo^ expression profile. Moving down the dendrogram, as indicated by dotted gray arrows, the ordered nodes split into 3 separate branches: an unpolarized (Ly6C^lo^CD206^lo^) trajectory, an M2-like (Ly6C^lo^CD206^hi^) trajectory, and a ‘hybrid’ (Ly6C^hi^CD206^hi^) macrophage trajectory (Fig. 5a, Supplementary Fig. 4a, b). Quantifying the number of macrophages within each annotated SPADE trajectory, it was found that there were no changes in the concentrations of M1-like or unpolarized macrophages between injury sizes at any timepoint. By contrast, both M2-like macrophages and hybrid macrophages were significantly elevated at 7 days post injury (Fig. 5b). Our data shows that CXCR4 expression elevates concurrently with CD206 for both M2-like and hybrid macrophage subtypes (Supplementary Fig. 4a). Thus, the dual expression of CD206 and CXCR4 may define specific macrophage subsets with a sustained and dysregulated response following critical VML.

**Fig. 6:**
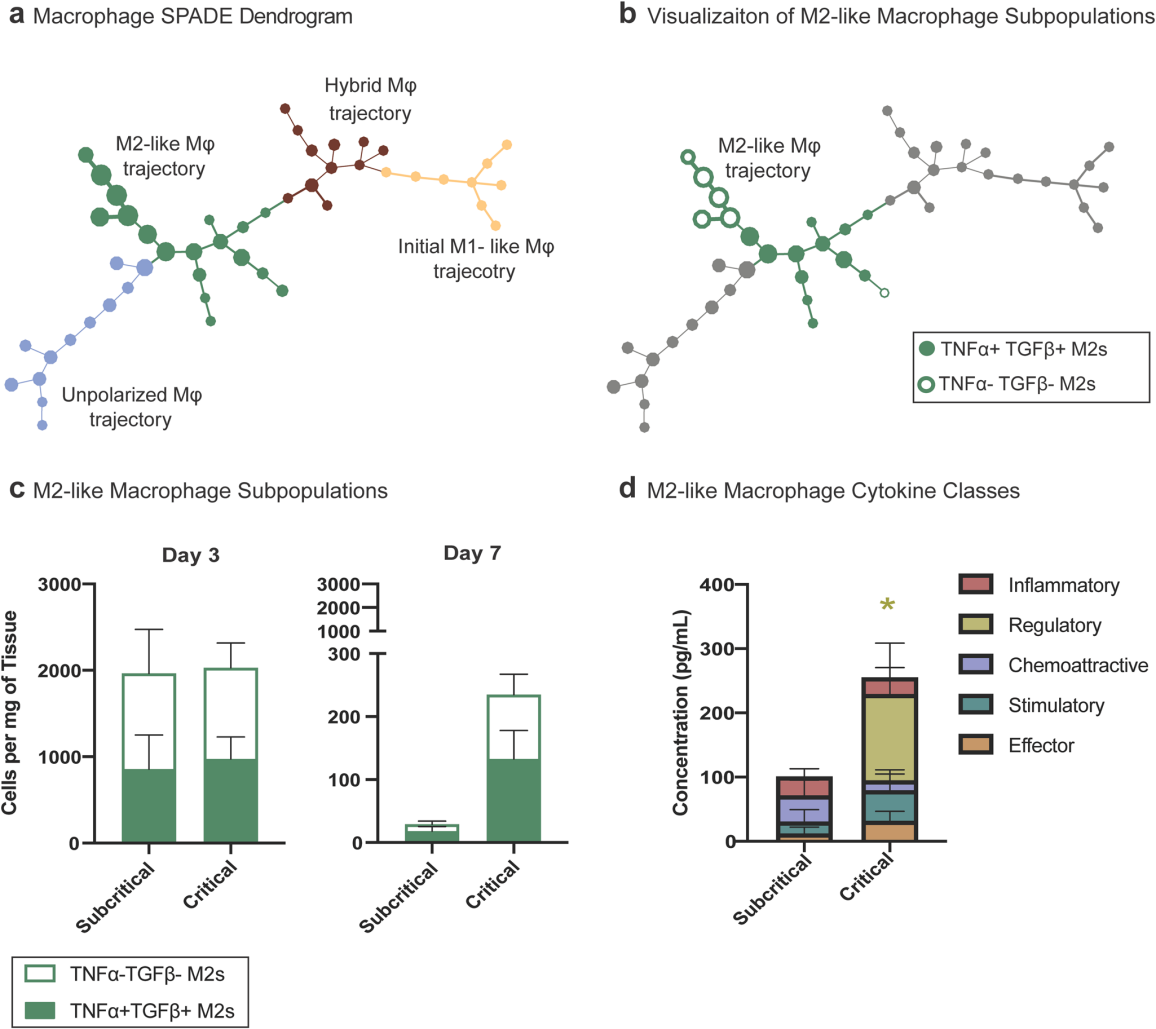
M2-like macrophages from critical VML defects have aberrant cytokine production profile that may contribute to pathological fibrosis. **a**–**c** Flow cytometry was performed on single cells isolated from quadriceps of mice that received a subcritical injury or critical VML injury at days 3 and 7 post injury. CD11b^+^CD64^+^MerTK^+^ macrophages pooled from all samples from both injury sizes and both timepoints were used to construct a SPADE dendrogram. **a** Macrophage SPADE dendrogram annotated based on CD206 and Ly6C expression to designate nodes to a M1-like, M2-like, unpolarized, or hybrid macrophage phenotype. **b** M2-like macrophages (green annotation) distinguished by their intracellular expression of TNF-α and TGF-β (TNF-α^+^ TGF-β^+^ nodes: solid green; TNF-α^-^ TGF-β^-^ nodes: green outline). **c** Number of TNF-α^+^ TGF-β^+^ and TNF-α^-^ TGF-β^-^ M2-like macrophages per mg of tissue represented as a stacked bar graph at day 3 and day 7 post subcritical injury or critical VML. **d** Concentration of cytokines (categorized into 5 classes) from lysate of FACS sorted M2-like macrophages at day 7 post subcritical injury and critical VML as measured by Isoplexis. Data presented as mean ± S.E.M. Statistical analysis included repeated measures two-way ANOVA with *Sidak* test between injury sizes per cytokine class. *p < 0.05 for regulatory cytokines between injury size. *n* = 3–4 (**a**–**c**) or 4 **d** biologically independent animals per experimental group. Mφ: macrophage.

While most studies have characterized the role of M2-like macrophages in establishing an anti-inflammatory microenvironment and promotion of tissue regeneration, in the presence of chronic inflammatory stimuli, M2-like macrophages are known to secrete large amounts of pro-fibrotic cytokines and promote tissue and organ fibrosis^36^. To examine the intracellular cytokine profile of the significantly elevated M2-like macrophages in critical VML, flow cytometry analysis was performed at days 3 and 7 post subcritical injury and critical VML. A SPADE dendrogram was constructed of all CD11b^+^CD64^+^MerTK^+^ macrophage events from both timepoints and injury sizes, and nodes were grouped into the same four phenotype subtypes (M1-like, M2-like, unpolarized, and hybrid) based on expression of Ly6C and CD206 (Fig. 6a, Supplementary Fig. 4c).

Within the M2-like macrophage subset, nodes were subsequently annotated based on expression of intracellular TNF-α and TGF-β. The M2-like macrophage trajectory segregated based on the expression profiles of these particular cytokines, indicating a divergence in M2-like macrophage phenotype progression as a function of injury severity. M2-like macrophage SPADE nodes that expressed both TNF-α and TGF-β were annotated as solid green whereas nodes that did not express either cytokine were annotated with only a green outline (Fig. 6b, Supplementary Fig. 4c). All nodes either expressed both or neither of the two cytokines, as there were no TNF-α^+^TGF-β^-^ or TNF-α^-^TGF-β^+^ expressing M2-like nodes. At day 3 post injury, there were no differences in the concentrations of TNF-α^+^TGF-β^+^ or TNF-α^-^TGF-β^-^ M2-like macrophages between injury sizes. At day 7, very few M2-like macrophages remain in subcritical defects, but there is a clear retention of M2-like macrophages in critical VML injured quadriceps- the majority of them expressing both TNF-α and TGF-β (represented as green segment of stacked bar graph) (Fig. 6c). To further probe this unique secretome, we harnessed multiplexed cytokine analysis to resolve functional heterogeneities of M2-like macrophages as a response to subcritical injury or critical VML. Sorted M2-like macrophages from critical VML had over a two-fold elevation in total cytokine production compared to subcritical injury. Interestingly, regulatory cytokines (IL4, IL10) specifically were significantly increased in critical VML, as they were undetected in subcritical injuries at the day 7 timepoint (Fig. 6d).

**Fig. 7:**
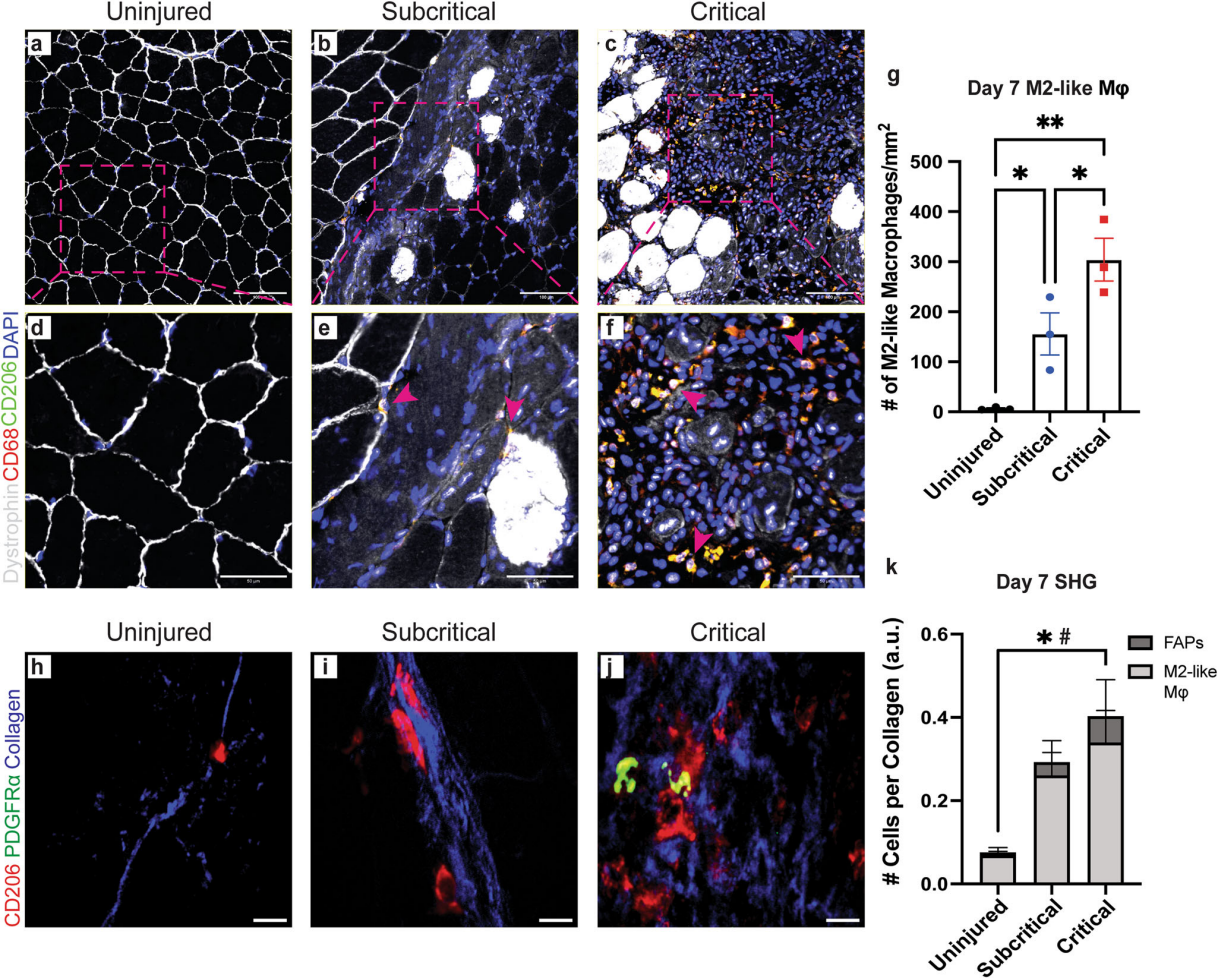
Retained M2-like macrophages in critical VML co-localize with FAPs in defect areas of marked collagen deposition. **a**–**f** Representative IHC images of quadriceps cross-sections from uninjured (**a**, **d**), subcritical injury (**b**, **e**), and critical VML (**c**, **f**) at 7 days post injury. Cross-sections stained for dystrophin (white), CD68 (red), CD206 (green), and DAPI (blue). Dotted pink boxes in **a**–**c** represent ROI regions presented in **d**–**f** where pink arrows indicate CD68^+^CD206^+^DAPI^+^ M2-like macrophages (yellow). **g** Quantified number of M2-like macrophages from each injury group at day 7 timepoint. **h**–**j** SHG imaging of representative day 7 cross-sections from uninjured (**h**), subcritical injury (**i**), and critical VML (**j**) quadriceps. Cross-sections stained for CD206 (red; M2-like macrophages) and PDGFRα (green; FAPs) with detected SHG signal (blue; collagen). **k** Number of M2-like macrophages (light gray) and FAPs (dark gray) per detected collagen signal within defect area imaged. Scale bars represent 100 µm (**a**–**c**), 50 µm (**d**–**f**), and 10 µm (**h**–**j**). Data presented as mean ± S.E.M. Statistical analyses performed include one-way ANOVA and repeated measures two-way ANOVA with *Holm-Sidak* test or Fisher’s LSD test, respectively. *p < 0.05 for number of M2-like macrophages per collagen, #*p* < 0.05 for number of FAPs per collagen. *n* = 3 biologically independent animals per group. IHC: immunohistochemistry; SHG: second harmonic generation; PDGFRα: platelet derived growth factor receptor alpha; FAPs: fibroadipogenic progenitors.

Uninjured, subcritical injury, and critical VML quadriceps cross-sections were used for immunohistochemical (IHC) analysis at 7 days post injury (Fig. 7) to visually assess the sustained presence of M2-like macrophages within the defect. Cross-sections were stained with dystrophin (white), DAPI (blue), CD68 (red) and CD206 (green), and M2-like macrophages were identified as CD68^+^CD206^+^DAPI^+^ (merged to yellow) cells within the muscle tissue. Uninjured control quadriceps showed healthy skeletal muscle morphology with DAPI^+^ myonuclei located at the periphery and little to no M2-like macrophages (Fig. 7a, d, g). Quadriceps that received subcritical injuries presented with visible DAPI^+^ cellular infiltration at day 7, some of which were identified as M2-like macrophages (Fig. 7b, e, pink arrows). The morphology of myofibers adjacent to the defect space appear largely unaffected. (Fig. 7b). In contrast, there was a significant increase in the infiltration and persistence of M2-like macrophages within the defect area of critically injured quadriceps (Fig. 7c, f, g, pink arrows) compared to both subcritical injury and uninjured tissue. There is an obvious ablation of tissue structure with necrotic myofibers spaced apart, distorted in their morphology, and with clusters of mononuclear cells surrounding them (Fig. 7c, f).

To explore whether retained M2-like macrophages with divergent cytokine profiles from critical VML are taking part in fibrotic signaling with FAPs, we performed second harmonic generation imaging on quadriceps sections stained for M2-like macrophages (CD206, red) and FAPs (PDGFRα, red) with collagen deposition illustrated in blue (Fig. 7h–j). Uninjured tissue presented with very little M2-like macrophages or FAPs localized near collagen (Fig. 7h). Critical VML resulted in significantly increased numbers of M2-like macrophages and FAPs per collagen within the injury site compared to uninjured tissue, whereas no differences were observed between uninjured and subcritical injury (Fig. 7k). Importantly, M2-like macrophages and FAPs co-localize in regions of collagen deposition in critical VML, but this was not often observed in subcritical defects (Fig. 7i, j).

### Chronic inflammatory stimuli from critical VML propagates unchecked T cell activation and recruitment to injury milieu

**Fig. 8:**
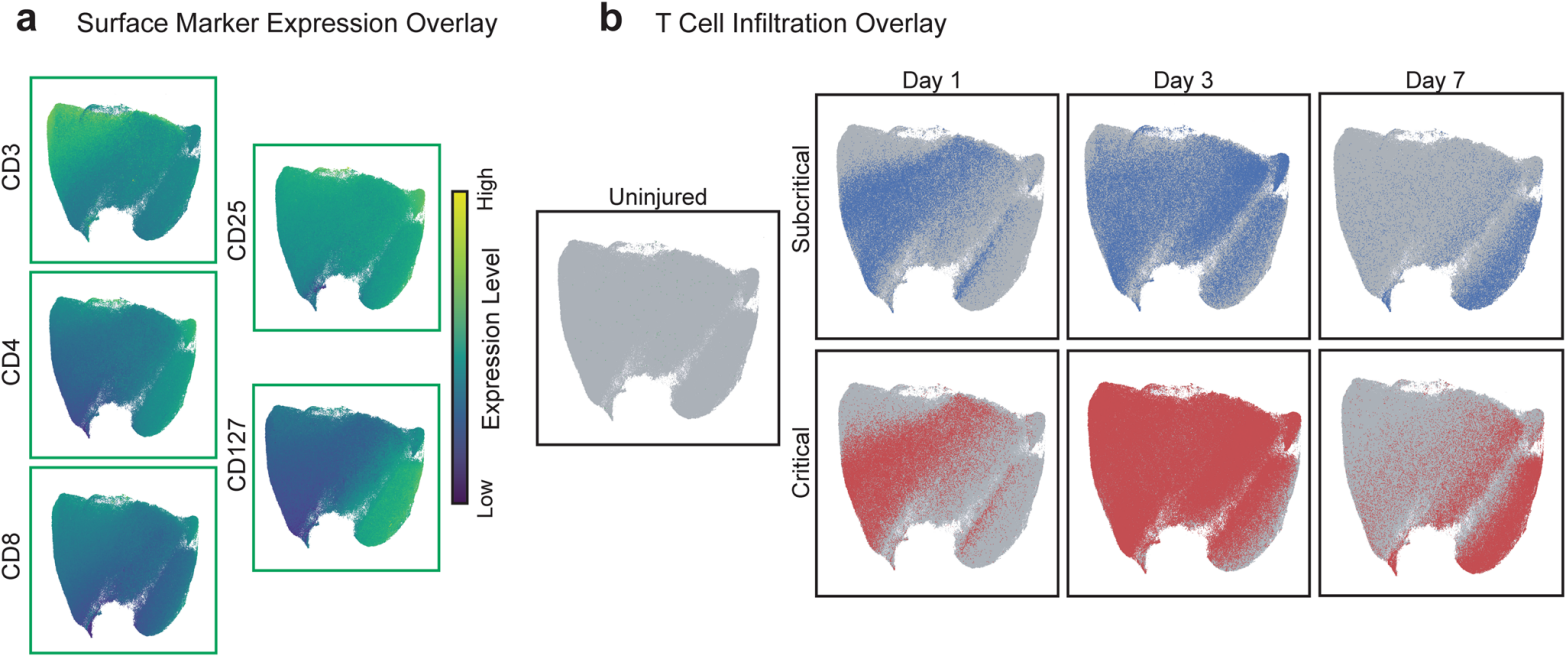
Critical VML injury presents with increased T cell recruitment 3 days post injury as visualized by UMAP. CD3^+^ T cells pooled from all quadricep samples (uninjured, subcritical injury, and critical VML) and all timepoints (days 1, 3, and 7) were used to generate a UMAP projection. **a** Expression levels of T cell surface markers overlaid onto UMAP. Marker expression levels range from dark blue to yellow, representing low to high expression, respectively. **b** CD3^+^ T cells isolated from uninjured quadriceps (green), subcritical injuries (blue, top), and critically sized injuries (red, bottom) at each timepoint. UMAP constructed from *n* = 4 biologically independent animals per experimental group.

All CD3^+^ T cells from all animals and at all timepoints (days 1, 3, and 7; bi-plot gating strategy of T cells in Supplementary Fig. 5) were used to construct a UMAP plot to visualize T cell infiltration and phenotype transitions in a lower dimensional space (Fig. 8). Relative expression levels of each measured T cell surface marker were overlaid onto the UMAP to illustrate the locations of heterogeneous T cell subpopulations within the UMAP space (Fig. 8a). The few T cells present in uninjured quadriceps were overlaid in green on the UMAP while T cells from subcritical injured quadriceps are overlaid in blue, and T cells from critical VML quadriceps are overlaid in red, by timepoint (Fig. 8b). The location of overlaid T cells from different biological samples provides a visual representation of T cell subtype dynamics following subcritical injury and critical VML.

Temporal dynamics of T cells were observed through the UMAP, as the left and right side of the UMAP projection is enriched for day 1 or day 7 T cells, respectively (Fig. 8b). T cells from subcritical injury and critical VML share similar surface marker dynamics, indicated by their similar location within the UMAP at each timepoint. At day 1, it appears that T cells have a low expression of CD4, expressed by helper T cells, and IL-7 receptor, CD127. Yet by day 3, there is a clear shift in phenotype as T cells localize in areas of high CD4 and CD127 expression, with T_reg_ marker CD25 and cytotoxic T cell marker CD8 remaining relatively constant with time (Fig. 8a, b). Importantly, increased T cell numbers from critical VML at days 3 and 7 post injury can be qualitatively observed in the UMAPs relative to subcritical injury (Fig. 8b) which may indicate hyperactivation of the adaptive immune response.

**Fig. 9:**
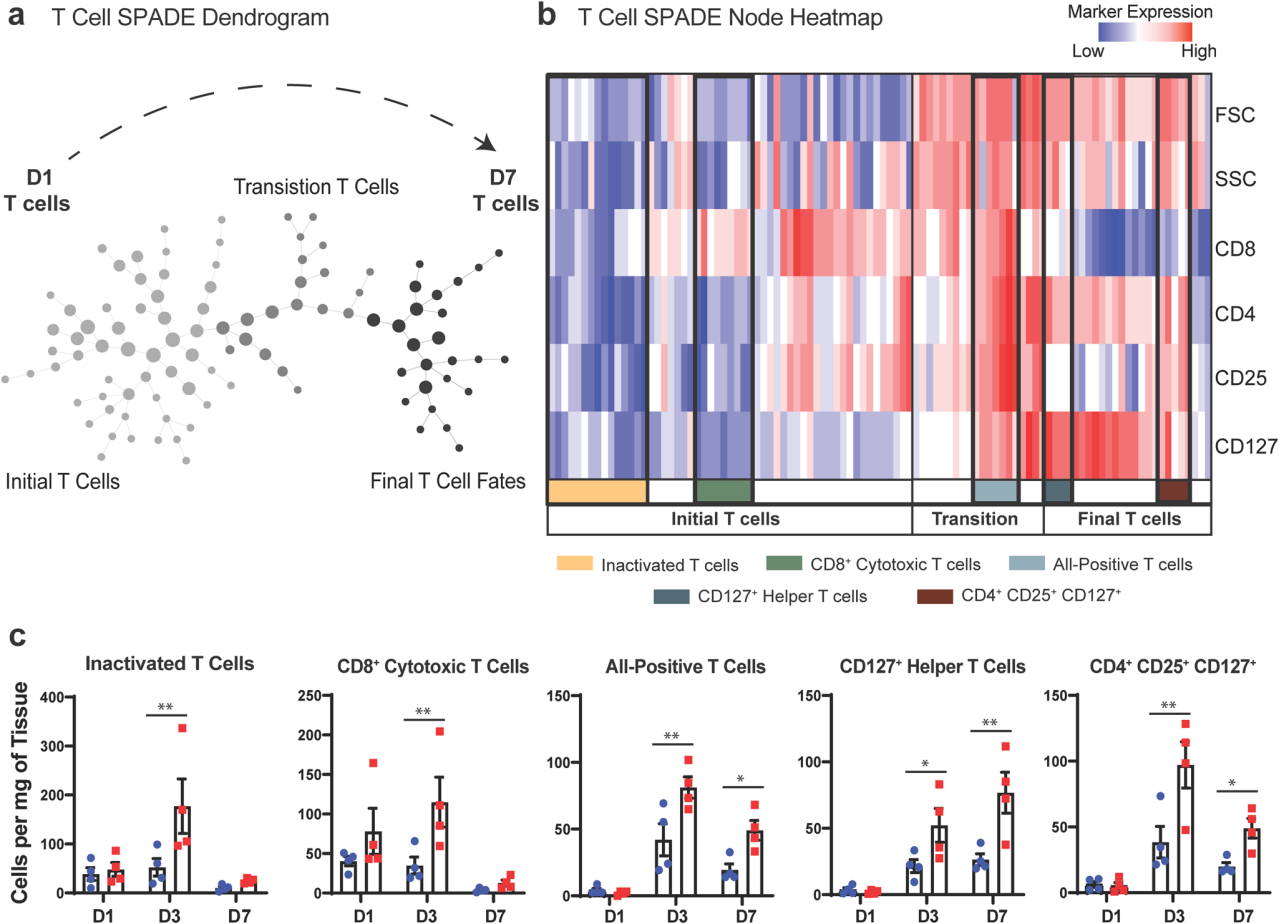
Pseudotime analysis reveals a dysregulated T cell response to critical VML injury. **a** Pre-gated CD3^+^ T cells from all samples (uninjured, subcritical injury, and critical VML) and all timepoints (days 1, 3, and 7) were used to construct a SPADE dendrogram. SPADE nodes annotated by relative percentage of T cells present from each timepoint. Gray arrow indicates time response of T cells with day 1 T-cells primarily occupying left side of dendrogram (light gray) and day 7 T cells primarily occupying nodes on right side of dendrogram (black). **b** Protein signature of each SPADE node represented as a heatmap. SPADE nodes grouped by temporal infiltration kinetics (initial T cells, transition T cells, or final T cell fates) and further annotated by FSC, SSC, and surface marker expression profiles. **c** T cell subtype cell counts quantified as cell concentration (cells/mg tissue). Quantified subtypes identified and annotated in **b**. Data presented as mean ± S.E.M. Statistical analyses performed on log transformed data with two-way ANOVA and *Sidak* multiple comparisons to determine differences between injury sizes. *p < 0.05, **p < 0.01, ****p < 0.0001, *n* = 4 biologically independent animals per experimental group. D1: day 1, D3: day 3, D7: day 7.

To characterize and quantify unique subsets of T cells within subcritical injury and critical VML, all CD3^+^ T cells from all animals and timepoints were used to generate a SPADE dendrogram (Fig. 9a). SPADE clustering of T cell events by their surface marker expression profile (Fig. 9b) also revealed a clear localization of T cells by timepoint indicating the phenotypic transition that occurs within the first week following injury. The nodes were grouped into 3 time-associated clusters: the initial T cell response (annotated light gray), transition T cells (annotated dark gray), and final T cell fates (annotated black) (Fig. 9a) as indicated by relative cell frequencies overlaid onto each SPADE node by timepoint (Supplementary Fig. 6a). The percentage of total T cells within each of these time-associated clusters was quantified (Supplementary Fig. 6b–d). The percentage of total T cells from day 1 samples in the initial T cell response population was significantly higher than the percentage of T cells from 3 and 7 days post injury, and the percentage of day 7 T cells is significantly lower than those at day 3 (Supplementary Fig. 6b). This result verifies that the left side of the SPADE dendrogram (light gray annotation) is comprised predominately of day 1 T cells. Within the transitional T cell population, representing a group of T cells that are not yet in their ‘final’ phenotype state, the percentage of T cells from days 3 and 7 were significantly higher than those from day 1 (Supplementary Fig. 6c). In final T cell fate nodes (far right of dendrogram), the percentage of T cells at day 7 were significantly elevated compared to the percentage at days 1 and 3 (Supplementary Fig. 6d). These findings confirm that the clustering of T cells via surface marker expression coincides with the progression of time (Fig. 9a, indicated with dashed black arrow). No differences were observed in the percentage of T cells between subcritical injury and critical VML at any time point within these time-associated node clusters.

Within each of the three time-associated node clusters, specific T cell subtypes of interest were identified by their unique marker expression profile as shown at each SPADE node in the form of a heatmap (Fig. 9b) or overlaid onto each node in the dendrogram (Supplementary Fig. 7). The SPADE nodes of the heatmap are organized from left to right in the same progression as the dendrogram. Within the greater ‘initial T cell response’ grouping, inactivated T cells were characterized by their low expression of all measured markers in addition to low forward scatter area (FSC) and side scatter area (SSC), which indicate small relative cell size and low intracellular granularity, respectively. The cells present in these nodes were quantified and it was found that there were significantly more inactivated T cells in critical VML at 3 days post injury compared to subcritical injuries (Fig. 9c). Nodes containing CD8^+^ cytotoxic T cells were identified by their high expression of CD8 but low expression of all other surface markers, FSC, and SSC (Fig. 9b). CD8^+^ cytotoxic T cells were significantly elevated at day 3 following critical VML injury (Fig. 9c).

As the dendrogram progresses into transition and final T cell fate stages, it is observed that cell size and intracellular granularity is increased as measured by FSC and SSC (Fig. 9b). A subpopulation of transitional T cells positive for all measured surface markers was identified. These ‘all-positive T cells’ were present within critically injured muscle at significantly higher concentrations at days 3 and 7 (Fig. 9b, c). Two subsets of interest were identified within the final fate T cells comprised chiefly of T cells from day 7. One of these subsets highly expressed both CD4 and CD127 (Fig. 9b, dark blue annotation). These CD127 + helper T cells were significantly increased at days 3 and 7 after critical VML (Fig. 9c). Finally, a group of nodes containing CD127^+^ T_reg_ cells (CD4^+^CD25^+^) were identified within the greater final fates T cells clustering. This T_reg_ subset presented with low expression of CD8 and was also significantly elevated at days 3 and 7 post injury in critical VML (Fig. 9b, c). Taken together, SPADE analysis was able to reconstruct the phenotypic transitions of T cell populations occurring through time and facilitated robust characterization and quantification of specific T cell subsets to reveal a dysregulated T cell response occurring at day 3 and 7 in critical VML.

## Discussion

The proper coordination of immune cells is crucial for prompt and proper regeneration and repair of damaged muscle following minor muscle injuries. Although skeletal muscle possesses remarkable regenerative capabilities, the traumatic loss of muscle characteristic of VML ablates the extracellular matrix and MuSC niche necessary for the initiation of myogenesis; thus, muscle’s innate capacity for regeneration becomes inadequate to functionally recover muscle^37^. In this series of studies, our data reveals key differences in the concentrations and temporal dynamics of identified immune cell subsets present within injured muscle following subcritical injury and critical VML. Subcritical injuries elicited an immune response similar to what is expected from an acute muscle injury, as most of the inflammation had been resolved by day 7. In contrast, critical VML presented with a sustained elevation of several unique myeloid and lymphoid cell subsets as characterized by UMAP and SPADE pseudo-time analysis. These results were summarized in Supplementary Fig. 8, where the average concentration data for each subtype and injury size were plotted to demonstrate the altered immune response to critical VML.

While single cell resolution by flow cytometry analysis provides a powerful tool for temporal evaluation of the immune response, increased dimensionality obscures the underlying heterogeneity of the data and increases the likelihood of introducing user bias when identifying cell populations^38^. Further, bi-plots only display correlations between two markers at a time, and it is difficult to fully characterize high-dimensional data with a series of two-dimensional visualizations. We harnessed the unbiased dimensionality reduction methods, UMAP and SPADE, to overcome these limitations and analyze the temporal progression of immune cells present in injured muscle dependent on injury size. We observed that critical VML injury showed increased CD11b^+^ cell retention in injured muscle at day 7 and a transient increase in MerTK, CXCR4 and CD206 expression. We found that in particular, mononuclear phagocytes elicited a prolonged immune response in critical VML, including a specific CD206^hi^Ly6C^lo^ subset that was not present in subcritical injuries by day 7, a finding that would not be easily discovered without implementation of advanced, dimensionality reduction visualization techniques.

We hypothesized that the sustained presence of mononuclear phagocytes within critical VML injuries may lead to the persistence of pro-inflammatory cytokines such as TNF-α and SDF-1. There was a significant increase in all identified monocyte subsets at day 3 post injury, suggesting an increased extravasation of monocytes from circulation to larger injuries. We found that at day 7 post injury, the concentrations of TNF-α^+^ neutrophils in addition to TNF-α^+^ and SDF-1^+^ monocytes were significantly elevated in critical VML but little to none were present in subcritical injuries. These results may indicate that early accumulation of pro-inflammatory myeloid cells in critical VML propagates the secretions of potent leukocyte chemo-attractants such as TNF-α and SDF-1, leading to immune cell retention at day 7 onwards. In an environment rich in cytokines such as TNF-α and IFN-γ, it is likely that MuSCs may efficiently activate and proliferate but fail to differentiate into myotubes as a result of a failed inflammatory-to-regenerative transition;^1,4^ thus, myogenesis is impaired and fibrotic and fatty infiltration fill the defect rather than functional muscle.

In minor injuries, elevation of M2-like macrophages and their progenitors, non-classical Ly6C^lo^ monocytes, has been linked with improved muscle healing^10,39^. However, our results indicate an abnormal persistence of M2-like macrophages in critical VML at day 7. Taking advantage of the unique capability of SPADE to preserve rare cell types often masked in bulk cellular analysis, we discovered a population of hybrid CD206 and Ly6C co-expressing macrophages which were also significantly increased in critical VML. We found that both of these CD206^hi^ macrophage subsets have concurrent elevations in CXCR4 expression which has been reported to be linked to fibrosis, in part through their expression and secretion of tissue inhibitor of metalloprotease 1 (TIMP1)^40^. It is notable that CXCR4 is elevated in M2s, as they are known to secrete pro-fibrotic cytokines such as TGF-β. It is also of interest that CXCR4 is highly expressed in hybrid macrophages, suggesting a potential role for CD206^hi^Ly6C^hi^ macrophages in fibrosis.

Previous studies have shown that macrophages simultaneously expressing pro- and anti-inflammatory cytokines may result from impaired M1-to-M2 phenotypic transitions and contribute to chronic inflammation and subsequent tissue fibrosis^6,41,42^. We sought to further characterize the cytokine profile of the M2-like macrophages persisting in critical VML and found that the majority of M2-like macrophages present at day 7 co-expressed TNF-α and TGF-β, as identified via SPADE clustering analysis. It has been shown that when macrophages co-express these cytokines, TGF-β dominates the response and leads to unregulated ECM deposition by FAPs to facilitate tissue fibrosis^6^. The hypothesis that aberrant M2-like macrophages induced by VML propagate pro-fibrotic signaling with FAPs is further evidenced by their increased co-localization with collagen in the injury milieu as captured by SHG imaging. Multiplexed cytokine analysis from sorted M2-like macrophages revealed significantly increased concentrations of IL-10 and IL-4 in critical VML relative to subcritical injury. While IL-10 is often considered a canonical anti-inflammatory cytokine, it has been found that over-exposure to IL-10 induced fibrocyte recruitment and exacerbated lung fibrosis;^43^ thus, its excess production in VML may exacerbate dysregulated ECM deposition. In our murine model, critical VML injured quadriceps resulted in increased vascular volume compared to uninjured tissue^24^, and M2-like macrophages have known roles for promoting angiogenesis in pathological conditions^44^. It is an interesting hypothesis that M2-like macrophages may be driving both the increased fibrotic and angiogenic response in VML, as fibroplasia and angiogenesis are known to be co-dependent processes in injury repair^45^. Future studies are necessary to elucidate the mechanisms by which M2-like macrophages regulate pro-fibrotic and pro-angiogenic responses within the injury milieu of VML.

T cells are known to play important roles in aiding macrophage trafficking and polarization during muscle healing^14,17^. Further, as phagocytic macrophages populate the injury, they present antigens on their cell surface and secrete cytokines responsible for activating T cells and the adaptive immune response^46^. We found that overall T cell numbers peaked around day 3 post injury, as would be expected^14^. Despite seemingly appropriate dynamics, we found that there were several subsets of T cells that presented with an altered response to critical VML at days 3 and 7 compared to subcritical injuries.

We qualitatively observed an increase in T cell size over time, represented by FSC, which indicates antigenic stimulation and T cell activation^47^. CD8^+^ cytotoxic T cells were increased at day 3 and a population of T cells highly expressing all measured markers was significantly elevated in critical VML at days 3 and 7, indicating increased T cell activation induced by larger injury size. CD4^+^CD8^+^ T cells have been shown to be highly cytotoxic which may lead to intensified tissue damage^48^, but further investigation is necessary to determine if these double-positive T cells are detrimental to muscle regeneration. Lastly, we identified two T cell populations expressing IL-7 receptor, CD127: CD127^+^CD4^+^ helper T cells and CD127^+^CD4^+^CD25^+^ T_reg_ cells. As CD127 has been reported to be expressed on activated T_reg_ cells in the presence of IL-7^49^, our results may be indicative of elevated IL-7 in our critical VML model. IL-7 has been found to reduce myoblast differentiation and fusion^50^, so future studies measuring whether increased IL-7 induced by critical VML upregulates CD127^+^ T cells and impedes myogenesis is of interest. While significant increases in T_reg_ cells after critical VML was not expected, it is possible that despite a local enrichment of T_reg_ cells, their immunosuppressive function is reduced in the presence of chronic inflammatory stimuli^51^. T cell exhaustion, a state of T cell dysregulation due to chronic antigen presentation^52^, could be impairing the role of T_reg_ cells to mediate myoblast differentiation during muscle regeneration. Often studied in the context of cancer and long-term infections, future studies evaluating the extent of T cell exhaustion occurring in severe trauma injuries such as VML would be greatly beneficial.

We have previously reported that 3 mm full thickness defects in the murine quadriceps VML model results in fibrosis, fatty infiltration, and lack of myofiber regeneration within the injury site- similar to the clinical scenario^24^. Here, we elucidate key immune cellular players that underlie this pathophysiology. Leveraging dimensionality reduction and clustering techniques to visualize the correlation of multiple measured markers across cell types was crucial to identifying altered cellular transitions at early timepoints after VML. However, these findings do not preclude that these SPADE-identified subsets could not be identified using more conventional strategies. One limitation to this series of studies is only assessing immune cell infiltration kinetics during the first week after injury. Uncovering cellular progressions thereafter and determining whether VML injured muscle reaches a verifiable state of resolution at a later timepoint will be a subject of future work. Revealing specific immune cell subtypes dysregulated in critical VML, particularly those with heterogeneous and progressive phenotypes, is an important approach to examining failed endogenous repair mechanisms at the cellular and molecular level. These studies may provide the necessary foundation for the development of targeted regenerative immunotherapies to improve clinical outcomes following VML.

## Methods

### Animals

C57BL/6 J mice were purchased from Jackson Laboratory and maintained as a breeding colony. All animals used in the study were male, 6.1 ± 0.5 (mean ± standard deviation) months in age at the time of euthanasia. A total of 60 mice were utilized for the presented series of studies.

### Quadriceps volumetric muscle loss injury

Surgical procedure performed as previously reported^24^. Briefly, the left hindlimb was prepped and sterilized. A single incision was made above the quadriceps and a 2 mm or 3 mm biopsy punch (VWR, 21909-132, -136) was used to make a full-thickness muscle defect. Skin was closed with wound clips and muscle was left to recover without intervention for 1, 3, or 7 days before euthanasia by CO_2_ inhalation. Naïve mice were utilized for uninjured control quadriceps. For all animals (injured mice or uninjured naïve mice), the left quadriceps muscle was dissected and analyzed.

### Tissue harvest and flow cytometry

Quadriceps were prepared for flow cytometry analysis on a FACS AriaIII flow cytometer (BD Biosciences) as previously reported^34^. Briefly, entire injured (or uninjured for controls), left quadriceps were harvested and digested with 5,500U/ml collagenase II and 2.5U/ml Dispase II for 1.5 h in a shaking 37 °C water bath. The digested muscles were filtered through a cell strainer to obtain a single cell suspension. Single-cell suspensions were stained for live cells using Zombie NIR (BioLegend, 1:100 dilution) dyes in cell-culture grade PBS per manufacturer instructions. Cells were then fixed in 4% PFA for 10 min at 4 °C. Cells were stained with cell phenotyping antibodies in a 1:1 volume ratio of 3% FBS and Brilliant Stain Buffer (BD Biosciences) according to standard procedures. The following antibodies were used in the T cell phenotyping panel: BV605-conjugated anti-CD4 (BioLegend), BV785-conjugated anti-CD8 (BioLegend), BV421-conjugated anti-CD3ε (BioLegend), PerCP-Cy5.5-conjugated anti-CD25 (BioLegend), and APC-conjugated anti-CD127 (BioLegend). The following antibodies were used for myeloid cell phenotyping: BV421 or PE-Cy5-conjugated anti-CD11b (BioLegend), APC-Cy7-conjugated anti-Ly6G, BV510 or PerCP-Cy5.5-conjugated anti-Ly6C (BioLegend), BV711 or FITC-conjugated anti-CD64 (BioLegend), PE or APC-conjugated anti-MerTK (BioLegend), PE-Cy7 conjugated anti-CD206 (BioLegend), FITC-conjugated anti-Ly6A/E (BioLegend), APC-conjugated Lineage antibody cocktail (BD Pharmigen), APC-conjugated anti-CD31 (BioLegend), PE-Cy5 conjugated anti-CD29 (BioLegend), and PerCP-Cy5.5-conjugated anti-CXCR4 (BioLegend). The following intracellular antibodies were used in flow cytometry experiments when indicated: BV510-conjugated anti-TNF-α (BioLegend), BV421-conjugated anti-TGF-β (BioLegend), and PE-conjugated anti-SDF-1 (R&D Systems). 30μL of CountBright Absolute Counting Beads (C36950, Invitrogen) were added per sample for absolute quantification of cell populations. All flow cytometry antibodies were used at a concentration of 0.25 or 0.5 µg per 100uL staining volume, in accordance with manufacturer recommendation.

### Immunophenotyping of myeloid and lymphoid cell subsets

Single, live cells were selected in FlowJo software for subsequent immunophenotyping analysis. Myeloid cells were identified as CD11b^+^ cells while T cell populations were identified as CD3^+^. Neutrophils were selected as CD11b^+^Ly6G^+^ cells. Monocytes were gated as CD11b^+^CD64^+^MerTK^-^SSC^lo^ cells and macrophages as CD11b^+^CD64^+^MerTK^+^ cells. Subsets of monocytes, macrophages, and T cells were further analyzed using SPADE analysis, as described. Conventional bi-plot gating strategies provided for all myeloid and lymphoid cell types and subpopulations (Supplementary Figs. 1–3, 5). A total of 28 quadriceps were used for myeloid and lymphoid immunophenotyping of uninjured and injured quadriceps at days 1, 3, and 7 post injury (*n* = 4 per experimental group). A total of 15 quadriceps were used for intracellular flow cytometry experiments performed at days 3 and 7 post injury (*n* = 3, 4 per experimental group).

### Uniform Manifold Approximation and Projection (UMAP)

UMAPs generated as previously reported^27,34,53^. Briefly, UMAP was used to embed high-dimensional flow cytometry data into a space of two dimensions, cells are visualized in a scatter plot where similarity is demonstrated via proximity to other points. Prior to UMAP dimensional reduction, each flow cytometry sample (*n* = 4 per experimental group) was pre-gated to select cellular subsets of interest (i.e. CD11b^+^ myeloid cells and CD3^+^ T cells) and then imported into Python 3.7 using fcsparser (https://github.com/eyurtsev/fcsparser) and Pandas 2.5. Each channel except for FSC and SSC was normalized by applying arcsinh/150. UMAP parameters of n_neighbors = 15 and min_dist = 0.1 were applied for compliance with UMAP assumptions. A composite UMAP projection that utilized data points from all desired samples was generated using Matplotlib. Cells from specific biological samples or timepoints were visualized by overlaying onto the generated UMAP projection which combined all samples and timepoints (https://github.com/lmcinnes/umap).

### Spanning-tree Progression Analysis of Density-normalized Events (SPADE)

SPADE trees generated as previously reported^27,34,53^. Briefly, SPADE was performed through MATLAB and the source code is available at http://pengqiu.gatech.edu/software/SPADE/. MATLAB-based SPADE automatically generates the tree by performing density-dependent down-sampling, agglomerative clustering, linking clusters with a minimum spanning-tree algorithm and up-sampling based on user input. The SPADE tree was generated by exporting uncompensated pre-gated live, single cells or select pre-gated cellular subsets (i.e. CD3^+^ T cells). The following SPADE parameters were used: Apply compensation matrix in FCS header, Arcsinh transformation with cofactor 150, neighborhood size 5, local density approximation factor 1.5, max allowable cells in pooled downsampled data 50000, target density 20000 cells remaining, and number of desired clusters 50-100, depending on cell population size.

### SPADE node heatmap

SPADE dendrogram heatmaps were constructed with calculated z-scores of fluorescence intensities for each measured surface marker across all nodes of a SPADE dendrogram. Each row of the heatmap corresponds to a surface marker and each column represents a single SPADE node. Marker expression levels range from dark blue to dark red, indicating low to high expression, respectively.

### Isoplexis cytokine secretome analysis

Quadriceps tissue from subcritical injury and critical VML injury were explanted at day 7 timepoint. Single cells were isolated and prepared for flow cytometry analysis as reported in “Tissue harvest and flow cytometry.” Single-cell suspensions were stained with the following antibodies: Zombie Red (1:100 dilution), APC-Cy7 conjugated anti-CD11b, FITC conjugated anti-CD64, APC conjugated anti- MerTK, PE-Cy7 conjugated anti-CD206, and PerCP-Cy5.5 conjugated anti-Ly6C. Single, live, CD11b^+^CD64^+^MerTK^+^ CD206^hi^Ly6C^lo^ M2-like macrophages were sorted on a FACS Aria IIIu flow cytometer (BD Biosciences). Sorted cells were prepared for multiplexed Isoplexis analysis as previously reported^54^. Briefly, 10,000 sorted M2-like macrophages from each animal (*n* = 4 per experimental group; 8 quadriceps total) were lysed using nondenaturing lysis buffer and centrifuged at 14,000xg for 10 min. Supernatant was collected and loaded into CodePlex chip according to manufacturer instructions. Lysis buffer was used for background measurements. Codeplex chips were inserted into IsoLight instrument to measure cytokine profile of each sample for all targeted cytokines (Isoplexis, CodePlex Mouse Adaptive Immune Panel). IsoSpeak software was used for automated quantitative measurements. Cytokines grouped by classes: Regulatory (IL-10, IL-4), Effector (IFN- γ, MIP-1a, TNF- α), Chemoattractive (IP-10, KC, RANTES), Stimulatory (GM-CSF, IL-12, IL-2, IL-5), Inflammatory (IL-17A, IL-1b, IL-6, MCP-1).

### Quadriceps tissue histology and immunostaining

Tissue processing and histology done as previously reported^24^. Briefly, muscle was dissected, weighed, and snap frozen in liquid nitrogen cooled isopentane. 10 µm cryosections (CryoStar NX70 Cryostat) were taken throughout the defect region. Samples were blocked and permeabilized before staining with anti-dystrophin (Abcam, ab15277, 1:200) and anti-CD68 (Abcam, ab53444, 1:150), diluted in blocking buffer, for 1-h incubation at room temperature. Secondary antibodies conjugated to Alexa Fluor 647 (Invitrogen, A21245, 1:250), Alexa Fluor 555 (Abcam, ab150158, 1:250), and Alexa Fluor 488- conjugated CD206 (BioLegend, 141710, 1:150) were incubated for 30 min at room temperature. Slides were mounted with Fluoroshield Mounting Medium with DAPI (Abcam, ab104139) and stored at 4 °C. Primary antibody anti-PDGFRα (Cell Signaling Technology, 3174, 1:200) was used for immunostaining samples prior to SHG imaging followed by staining with secondary antibody conjugated to Alexa Fluor 421 (Abcam, ab175652, 1:250) and Alexa Fluor 488-conjugated CD206 (BioLegend, 141710, 1:150). Slides prepared for SHG were mounted with PerMount Mounting Medium (VWR, 100496) and stored at 4 °C.

### Confocal imaging and quantification of M2-like macrophages in quadriceps cross-sections

Immunofluorescence images were taken on Nikon W1 Spinning Disk Confocal microscope at 20x and stitched together with Nikon NIS-Elements imaging software. The number of M2-like macrophages were quantified by taking five representative regions of each section for three replicate sections per animal (*n* = 3 animals per experimental group; 9 quadriceps total). The three sections per animal selected for analysis came from different locations within the defect: one from the proximal end of the defect, one in the center of the defect, and one taken at the distal end of the injury. CD68^+^CD206^+^DAPI^+^ M2-like macrophages were counted using the ImageJ multipoint tool and summed for all five representative regions per section. Total M2-like macrophage numbers from each of the three selected sections were averaged and normalized to total region of interest area.

### Second harmonic generation (SHG) imaging of quadriceps cross-sections and quantification of M2-like macrophages and FAPs per collagen

Quadriceps cryosections of 10 µm thickness were prepared and immunostained as described above. Multispectral imaging of the slide mounted quadricep muscles was performed using a custom multiphoton microscope, similar to the previously reported setup^55–57^. In short, the excitation source is a Ti:Sapphire femtosecond pulsed laser (Chameleon Ultra II, Coherent), set to output a wavelength of 775 nm, the power of which is adjusted by a half-waveplate and polarizing beam splitter, and is rapidly modulated by a Pockels cell. A combination resonant scanner and galvo scanner enables fast scanning of the excitation beam across the sample. Light emitted from the sample is collected by photon multiplier tubes, allowing for the capture of three spectral channels. Bandpass filters at 542 (targeting M2-like macrophages), 457 (targeting FAPs) and 390 (targeting collagen using SHG) provide the requisite specificity for single cell analysis. For each injury condition, ~15 images within a region of 80μm x 80μm of each of three samples were collected. Individual M2-like macrophages and FAPs were counted, while a measurement of collagen was performed by thresholding the SHG images using the default Otsu method in ImageJ. M2-like macrophages and FAP counts were divided by the mean value of the thresholded images (range 0-255) to provide a metric for cell count relative to collagen.

### Statistics and reproducibility

All statistical analyses were done in GraphPad Prism 8. Data displayed with outlined bars representing the mean, error bars are ± Standard Error of the Mean (S.E.M.). For multiple comparisons, one-way or two-way ANOVA, as appropriate, with *Sidak* test unless otherwise indicated*, p* < 0.05 considered significant. Statistical analyses utilized individual animals as biologically independent replicates. Flow cytometry gating, analysis, and histological quantifications were performed in a blinded manner.

### Study approval

All animal studies were approved by the Georgia Institute of Technology Institutional Animal Care and Use Committee.

### Reporting summary

Further information on research design is available in the Nature Portfolio Reporting Summary linked to this article.

## Supplementary information

Supplementary Figures

Description of Additional Supplementary Files

Supplementary Data 1

Reporting Summary

## Data Availability

The authors declare that all relevant data supporting the findings of this study are available within the paper and its supplementary files. All source data for main figures is provided in Supplementary Data 1. All other data will be available from corresponding authors upon reasonable request.

## Code availability

The analysis software used in this article is open source. The MATLAB source code for SPADE is available at http://pengqiu.gatech.edu/software/SPADE/. UMAP source code used in these studies are available through GitHub (https://github.com/lmcinnes/umap).
