# Supplementary material for: Identifying dysregulated immune cell subsets following volumetric muscle loss with pseudo-time trajectories
Source: Commun Biol. 2023 Jul 19;6:749. doi: 10.1038/s42003-023-04790-6 (PMC10356763; doi:10.1038/s42003-023-04790-6)
Supplement: Supplementary file 1 — Supplementary Figures [file 42003_2023_4790_MOESM1_ESM.pdf]

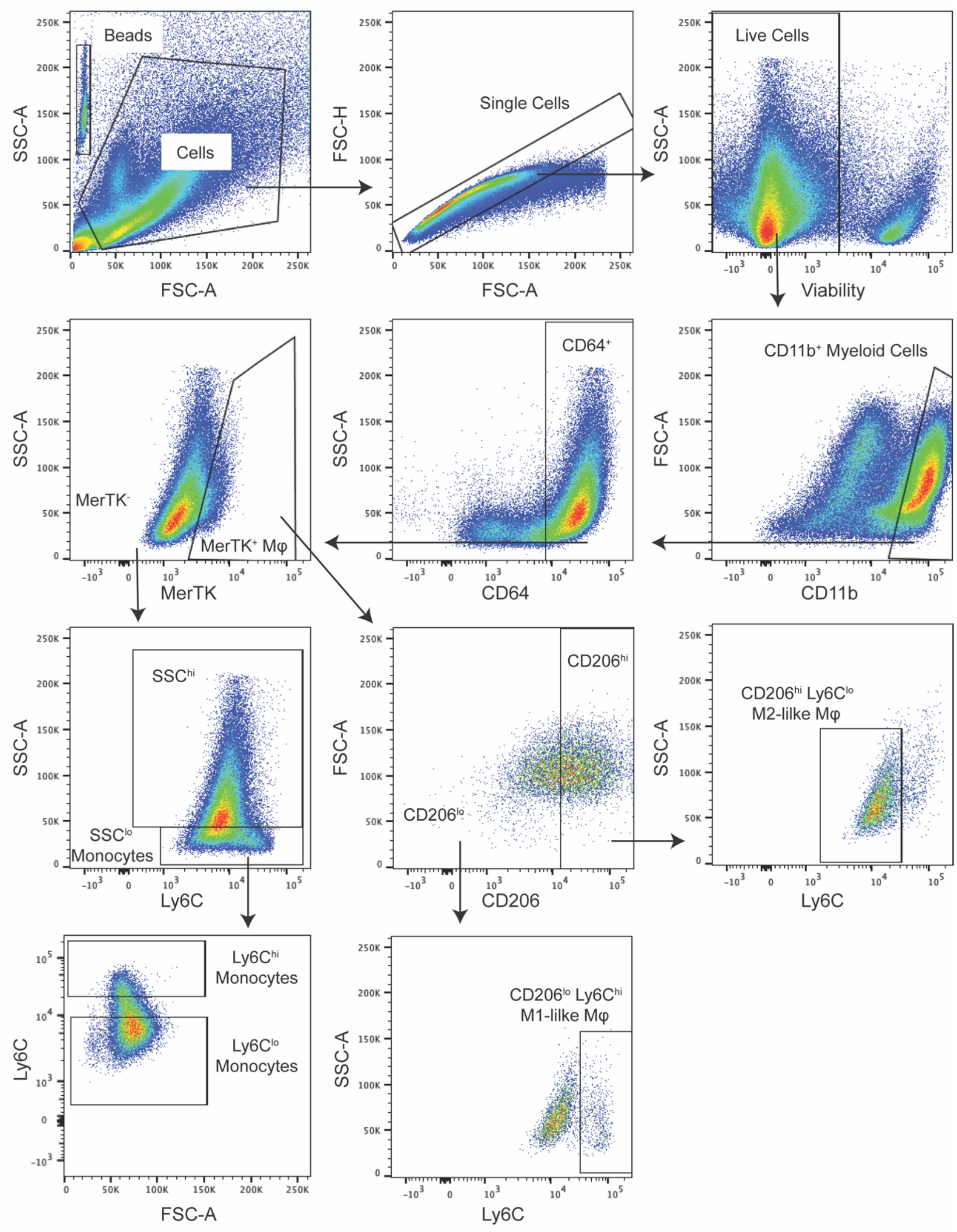

Supplementary Figure 1. Traditional gating strategy for myeloid cell immunophenotyping of single cells extracted from quadriceps tissue. Bi-plot gating strategy for myeloid cell immunophenotyping of single cells extracted from uninjured, subcritical injury, or critical VML injured quadriceps (days 1, 3, and 7 post injury) in FlowJo software. Cell phenotypes determined by expression of surface markers included in myeloid cell flow cytometry panel.

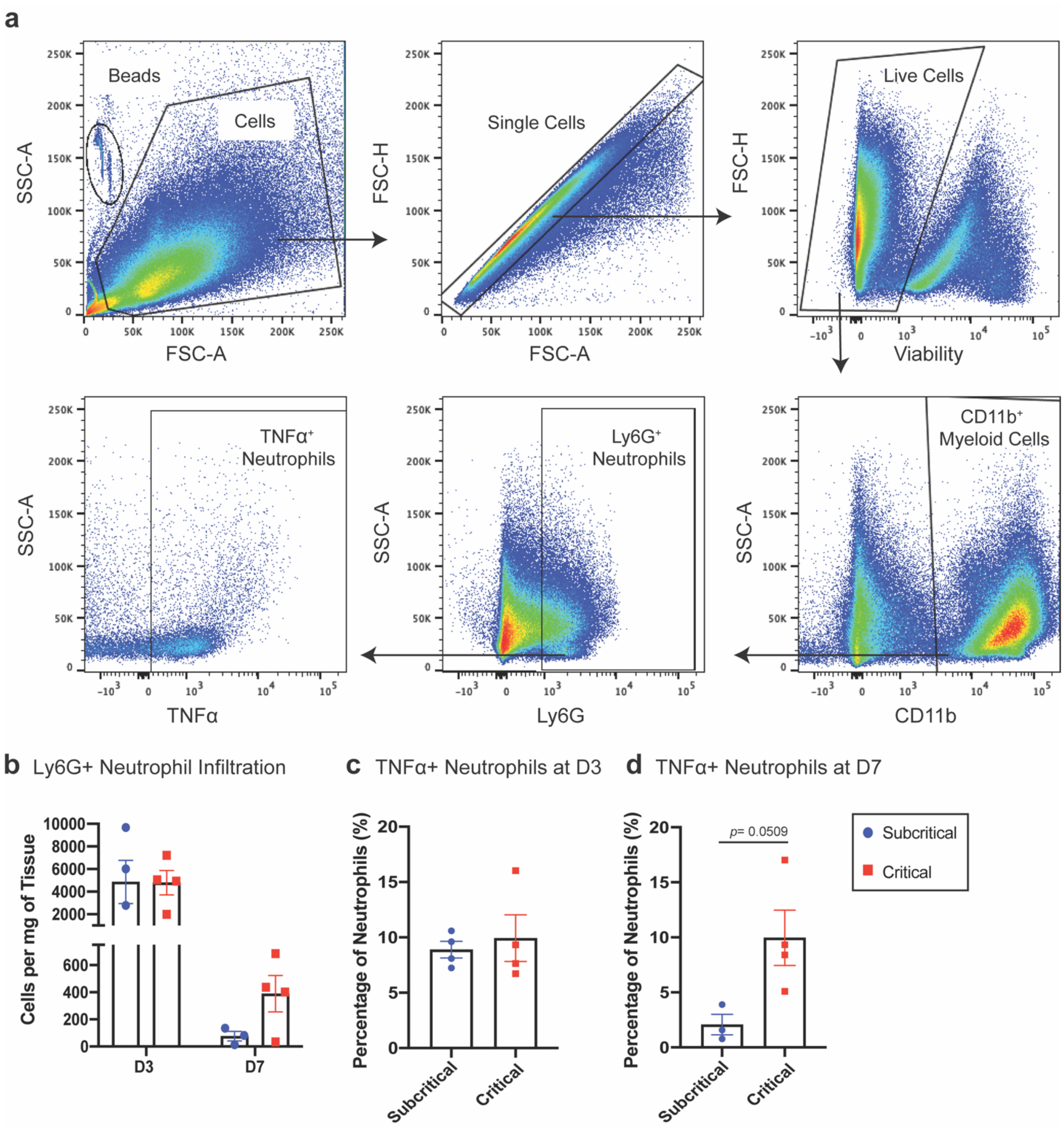

Supplementary Figure 2. TNF-α^{+} neutrophils persist within critically sized VML injuries at day 7. Single cell flow cytometric analysis performed at days 3 and 7 post subcritical injury or critical VML injury to murine quadriceps. **(a)** Bi-plot gating strategy of CD11b^{+}Ly6G^{+} neutrophils expressing TNF-α utilizing intracellular immunophenotyping flow cytometry panel. **(b)** CD11b^{+}Ly6G^{+} neutrophil concentration within injured quadriceps at each timepoint post injury. **(c)** The percentage of total neutrophils that express TNF-α from subcritical injury and critical VML at day 3. **(d)** The percentage of total neutrophils that express TNF-α from subcritical injury and critical VML at day 7. Data presented as mean ± S.E.M. Statistical analysis conducted with unpaired t-test**.** n= 3-4 biologically independent animals per experimental group. D3: day 3, D7: day 7.

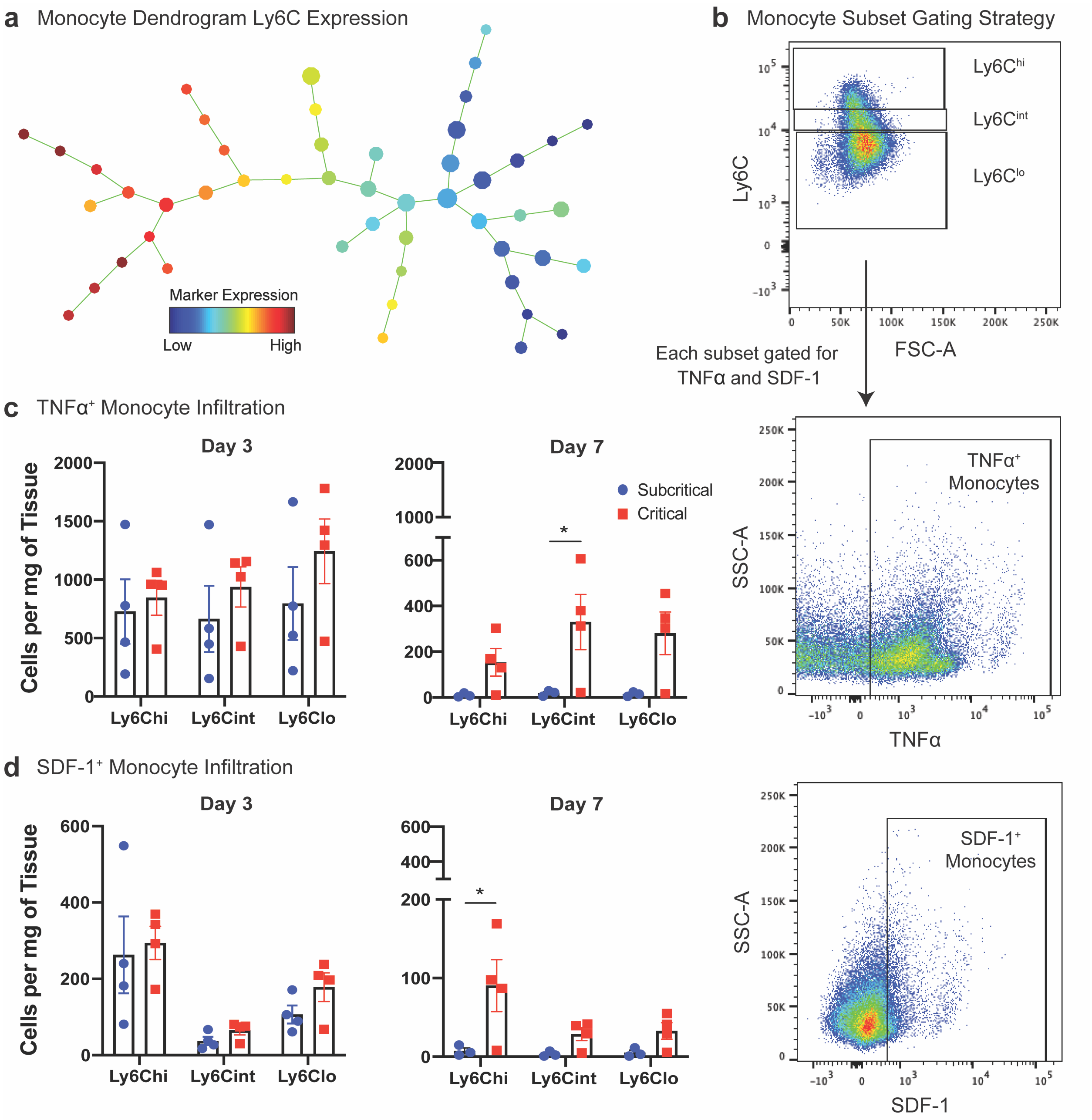

Supplementary Figure 3. Monocyte subsets infiltrating critically sized defects may propagate chronic inflammation via TNF-α and SDF-1 cytokine signaling. (a) Median Ly6C expression per node overlaid onto monocyte SPADE dendrogram to distinguish monocyte subsets (see Figure 4). Expression levels range from blue to red, representing low to high median expression, respectively. **(b-d)** Single cell flow cytometric analysis performed at days 3 and 7 post subcritical injury or critical VML to murine quadriceps to assess monocyte cytokine expression. **(b)** CD11b^{+}CD64^{+}MerTK^{-}SSC^{lo} monocytes characterized into Ly6C^{lo}, Ly6C^{int}, or Ly6C^{hi} subsets based on expression of Ly6C. Each subset was subsequently assessed for TNF-α and SDF-1. **(c)** Concentration of each monocyte subset expressing TNF-α at day 3 and 7 post subcritical injury or critical VML. **(d)** Concentration of each monocyte subset expressing SDF-1 at day 3 and 7 post subcritical injury or critical VML. Data presented as mean ± S.E.M. Statistical analyses include two-way ANOVA with *Sidak* multiple comparisons test between injury sizes. *$p<0.05$, n= 3-4 biologically independent animals per experimental group.

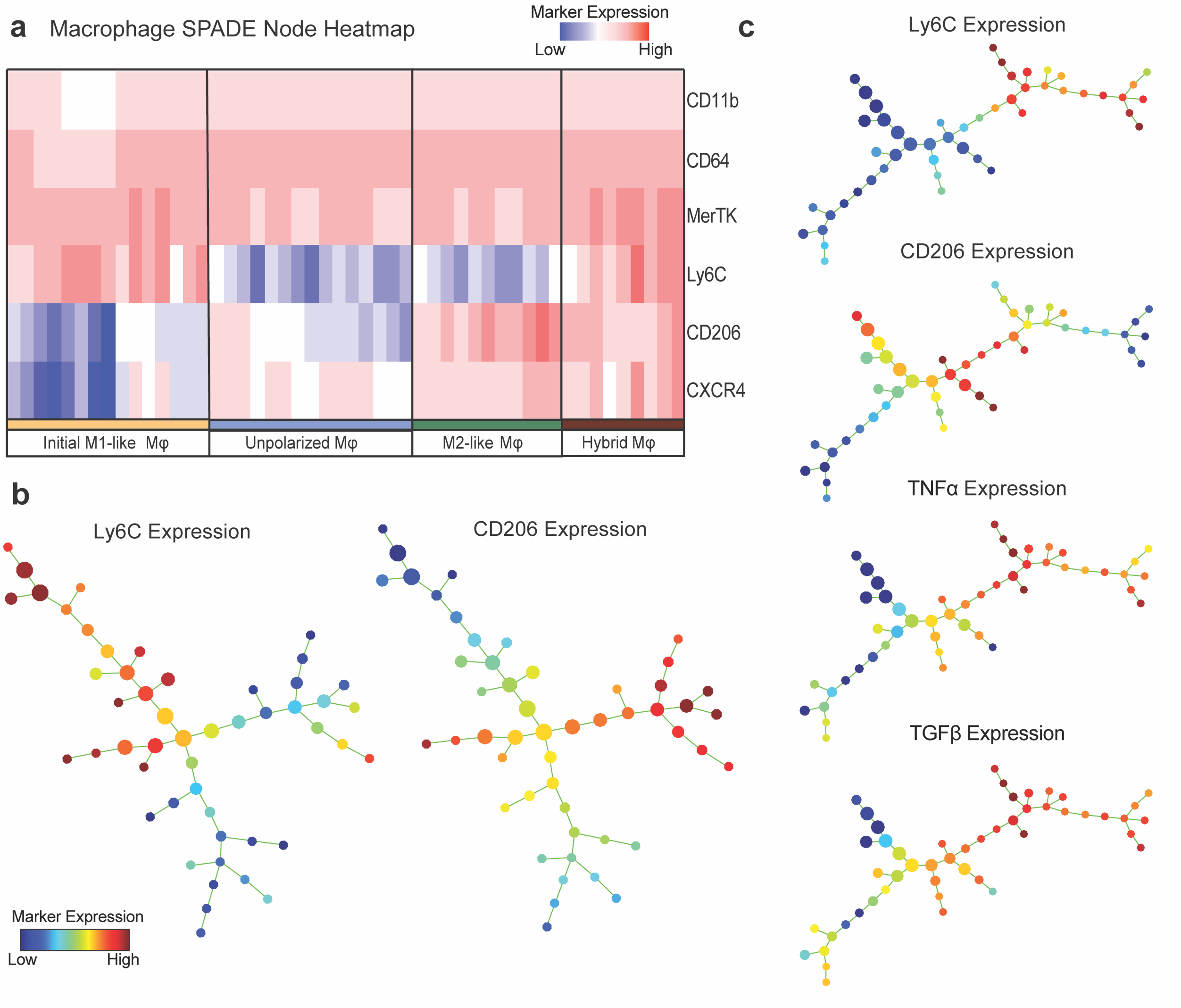

Supplementary Figure 4. Characterization of macrophage subset phenotypes visualized by SPADE node marker expression. (a) Heatmap rendering of marker expression levels within each macrophage SPADE node ranging from dark blue to dark red, representing low to high marker expression, respectively. Each column of the heatmap represents one SPADE node and each row is one surface marker as labeled. SPADE dendrogram was comprised of $CD11b^{+}CD64^{+}MerTK^{+}$ macrophages pooled from all injury groups (uninjured, subcritical injury, and critical VML) and timepoints (days 1, 3, and 7). **(b)** Median expression of Ly6C and CD206 overlaid onto each node of macrophage SPADE dendrogram (see Figure 5). **(c)** Median expression of Ly6C, CD206, TNF-α, and TGF-β overlaid onto each node of macrophage SPADE dendrogram constructed from intracellular cytokine flow panel (see Figure 6). Expression levels range from blue to red, representing low to high median expression, respectively.

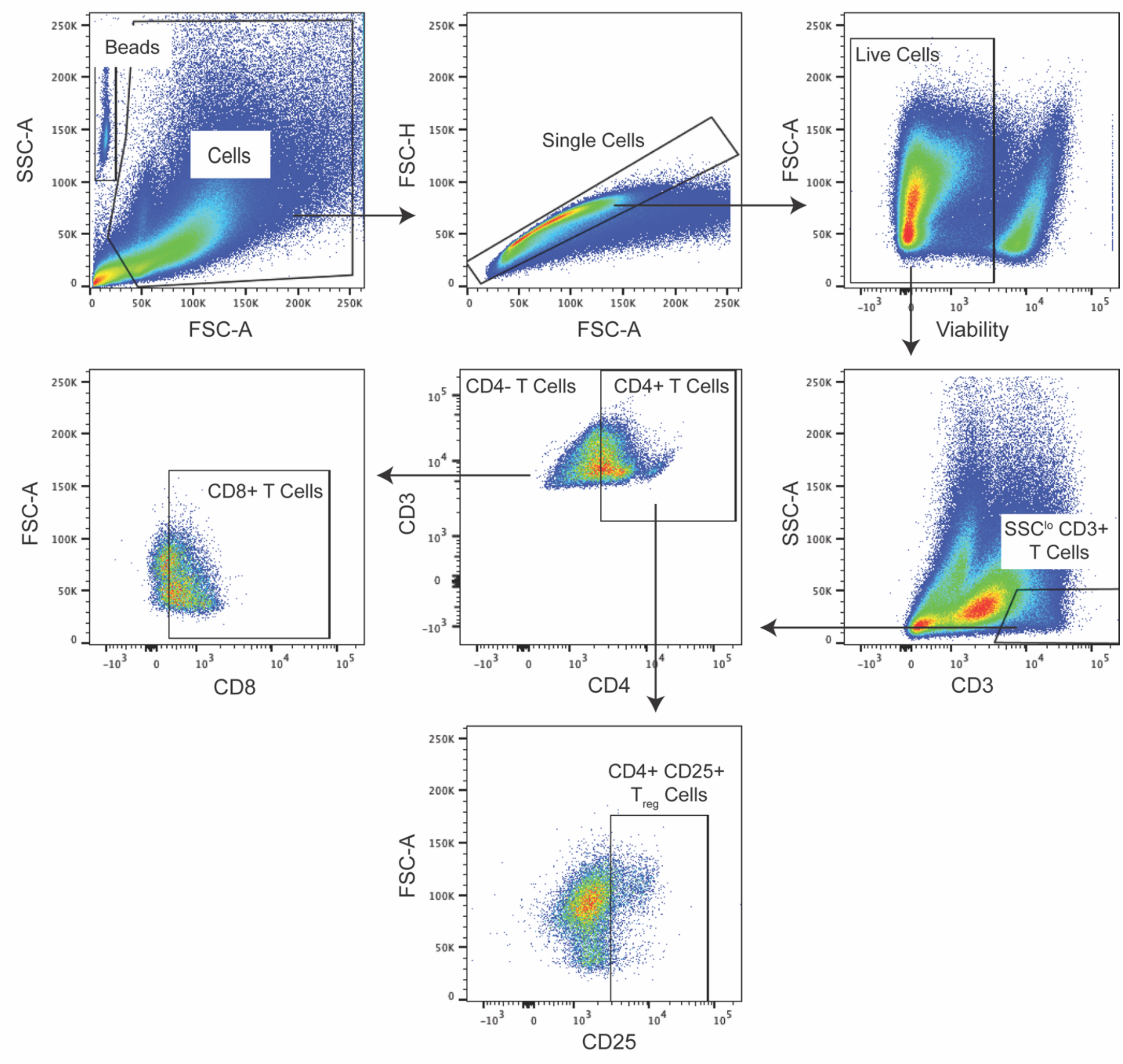

Supplementary Figure 5. Traditional gating strategy for T cell immunophenotyping of single cells extracted from quadriceps tissue. Bi-plot gating strategy for T cell immunophenotyping of single cells extracted from uninjured, subcritical injury, or critical VML quadriceps (days 1, 3, and 7 post injury) in FlowJo software. Cell phenotypes determined by expression of surface markers included in T cell flow cytometry panel.

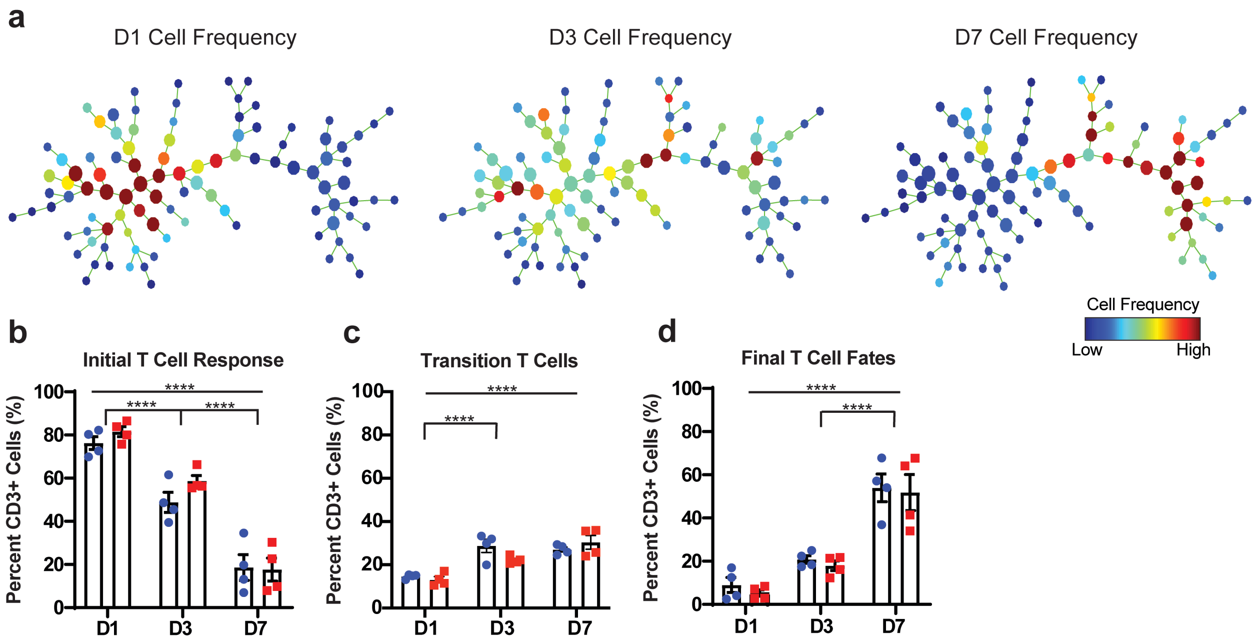

Supplementary Figure 6. Temporal infiltration of T cells from subcritical injury and critical VML. **(a)** SPADE dendrogram comprised of $CD3^+$ T cells from uninjured, subcritical injury, and critical VML quadriceps at all timepoints (days 1, 3, 7). SPADE nodes annotated by relative percentage of T cells present from each timepoint within the dendrogram. Cell frequency annotations ranges from blue to red, representing low to high cell frequency per node, respectively. **(b-d)** The percentage of total T cells from each timepoint within each annotated, temporally determined region of the SPADE dendrogram. Data presented as mean ± S.E.M. Statistical analyses performed as two-way ANOVA with *Sidak* multiple comparisons to determine differences between timepoints. *****p*<0.0001, n=4 biologically independent animals per experimental group. D1: day 1, D3: day 3, D7: day 7.

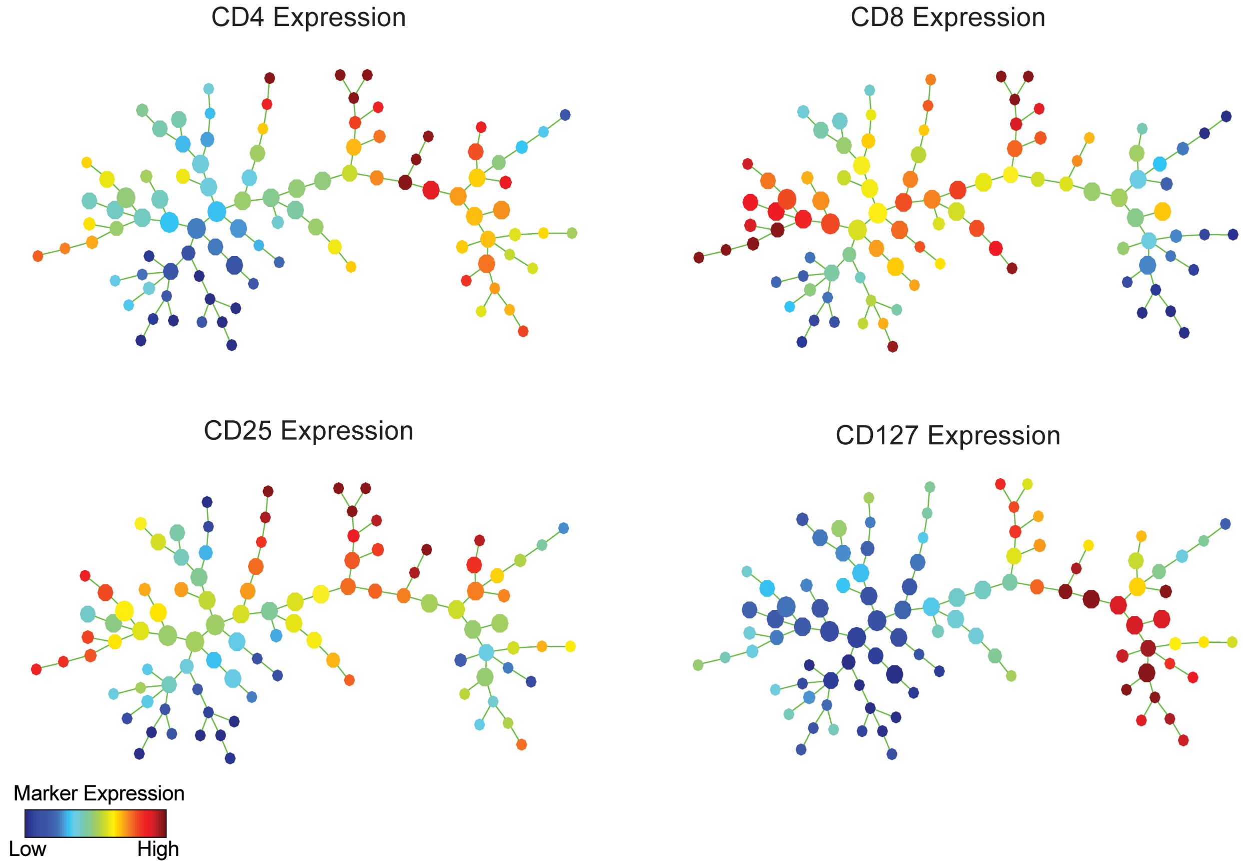

Supplementary Figure 7. Identification of T cell subsets via SPADE node marker expression. SPADE dendrogram constructed using pre-gated $CD3^+$ T cells extracted from uninjured, subcritical injury, and critical VML quadriceps at all timepoints (days 1, 3, 7). Median surface marker expression of CD4, CD8, CD25, and CD127 overlaid onto each node of T cell SPADE dendrogram to distinguish T cell subpopulations. Expression annotations range from blue to red, indicating low to high median expression, respectively.

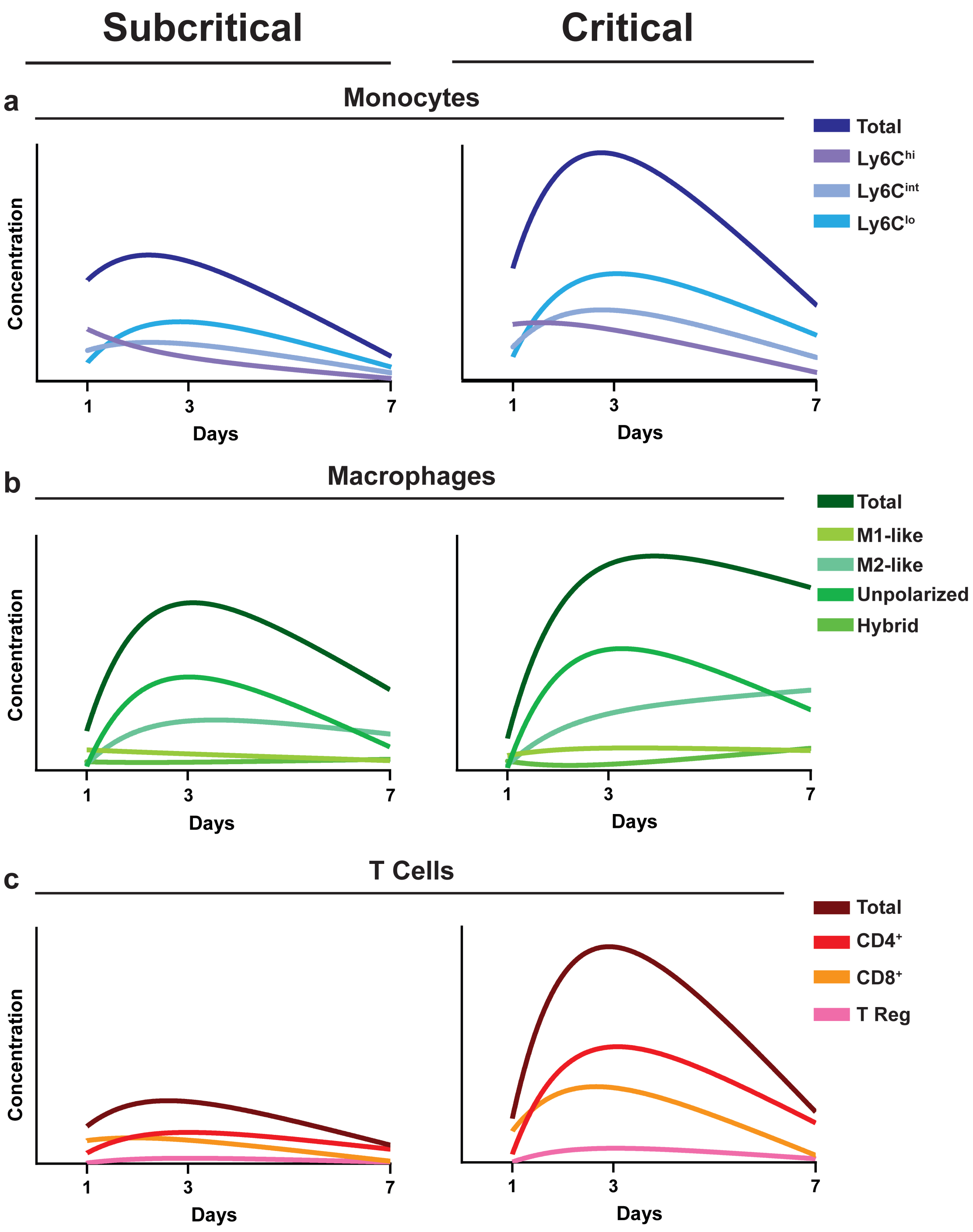

Supplementary Figure 8. Temporal evaluation of immune cell response to subcritical injury and critical VML. Summary of the immune cell response in the first week following subcritical injury and critical VML. Cell subpopulation dynamics from each injury size represented as lines fit with a continuous hinge function (GraphPad Prism 8) through the quantified concentration data for monocytes **(a)**, macrophages **(b)**, and T cells **(c)**. Axes values were kept consistent between cell populations.
