# Supplementary material for: Identifying dysregulated immune cell subsets following volumetric muscle loss with pseudo-time trajectories
Source: Commun Biol. 2023 Jul 19;6:749. doi: 10.1038/s42003-023-04790-6 (PMC10356763; doi:10.1038/s42003-023-04790-6)
Supplement: Supplementary file 2 — Description of Additional Supplementary Files [file 42003_2023_4790_MOESM2_ESM.pdf]

Description of Additional Supplementary Files

File name: Supplementary Data
Description: The source data behind the graphs presented in the main text figures.
