# Supplementary material for: Identifying dysregulated immune cell subsets following volumetric muscle loss with pseudo-time trajectories
Source: Commun Biol. 2023 Jul 19;6:749. doi: 10.1038/s42003-023-04790-6 (PMC10356763; doi:10.1038/s42003-023-04790-6)
Supplement: Supplementary file 4 — Reporting Summary [file 42003_2023_4790_MOESM4_ESM.pdf]

nature portfolio

Corresponding author(s): Edward A. Botchwey, Nick J. Willet, Young C. Jang

Last updated by author(s): March 19, 2023

Reporting Summary

Nature Portfolio wishes to improve the reproducibility of the work that we publish. This form provides structure for consistency and transparency in reporting. For further information on Nature Portfolio policies, see our Editorial Policies and the Editorial Policy Checklist.

Statistics

For all statistical analyses, confirm that the following items are present in the figure legend, table legend, main text, or Methods section.

n/a	Confirmed	
☐	☒	The exact sample size (*n*) for each experimental group/condition, given as a discrete number and unit of measurement
☐	☒	A statement on whether measurements were taken from distinct samples or whether the same sample was measured repeatedly
☐	☒	The statistical test(s) used AND whether they are one- or two-sided<br>*Only common tests should be described solely by name; describe more complex techniques in the Methods section.*
☒	☐	A description of all covariates tested
☐	☒	A description of any assumptions or corrections, such as tests of normality and adjustment for multiple comparisons
☐	☒	A full description of the statistical parameters including central tendency (e.g. means) or other basic estimates (e.g. regression coefficient) AND variation (e.g. standard deviation) or associated estimates of uncertainty (e.g. confidence intervals)
☐	☒	For null hypothesis testing, the test statistic (e.g. *F*, *t*, *r*) with confidence intervals, effect sizes, degrees of freedom and *P* value noted<br>*Give P values as exact values whenever suitable.*
☒	☐	For Bayesian analysis, information on the choice of priors and Markov chain Monte Carlo settings
☒	☐	For hierarchical and complex designs, identification of the appropriate level for tests and full reporting of outcomes
☒	☐	Estimates of effect sizes (e.g. Cohen's *d*, Pearson's *r*), indicating how they were calculated

Our web collection on statistics for biologists contains articles on many of the points above.

Software and code

Policy information about availability of computer code

Data collection	Flow cytometry and cell sorting was performed on a FACS AriaIII flow cytometer (BD Biosciences). Cytokine data was collected on an IsoLight instrument with CodePlex chips (Isoplexis). Microscope slides of tissue cryosections were fluorescently imaged on a Nikon W1 Spinning Disk Confocal microscope at 20x. Second harmonic generation imaging of tissue sections was performed on a custom multiphoton microscope (citations provided in manuscript describing microscope setup).
Data analysis	All statistical and graphical analysis was performed in GraphPad Prism 8. FlowJo software was used to gate flow cytometry data. SPADE and UMAP analysis was performed on the flow cytometry data presented in this research article for visualization and clustering analysis. All computer code is open source with links provided in code availability section of manuscript. IsoSpeak software (Isoplexis) was used for automated quantitative measurements for cytokine multiplexing of sorted M2-like macrophages. Nikon NIS-Elements imaging software and ImageJ were utilized for image analysis.

For manuscripts utilizing custom algorithms or software that are central to the research but not yet described in published literature, software must be made available to editors and reviewers. We strongly encourage code deposition in a community repository (e.g. GitHub). See the Nature Portfolio guidelines for submitting code & software for further information.

Data

Policy information about availability of data

All manuscripts must include a data availability statement. This statement should provide the following information, where applicable:

- Accession codes, unique identifiers, or web links for publicly available datasets
- A description of any restrictions on data availability
- For clinical datasets or third party data, please ensure that the statement adheres to our policy

The authors declare that all relevant data supporting the findings of this study are available within the paper and its supplementary files. All source data for main figures is provided in Supplementary Data 1. All other data will be available from corresponding authors upon reasonable request.

Human research participants

Policy information about studies involving human research participants and Sex and Gender in Research.

Reporting on sex and gender	Human research N/A in this manuscript.
Population characteristics	*Describe the covariate-relevant population characteristics of the human research participants (e.g. age, genotypic information, past and current diagnosis and treatment categories). If you filled out the behavioural & social sciences study design questions and have nothing to add here, write "See above."*
Recruitment	*Describe how participants were recruited. Outline any potential self-selection bias or other biases that may be present and how these are likely to impact results.*
Ethics oversight	*Identify the organization(s) that approved the study protocol.*

Note that full information on the approval of the study protocol must also be provided in the manuscript.

Field-specific reporting

Please select the one below that is the best fit for your research. If you are not sure, read the appropriate sections before making your selection.

☒ Life sciences ☐ Behavioural & social sciences ☐ Ecological, evolutionary & environmental sciences

For a reference copy of the document with all sections, see nature.com/documents/nr-reporting-summary-flat.pdf

Life sciences study design

All studies must disclose on these points even when the disclosure is negative.

Sample size	Sample sizes were determined based on the minimum number of animals required to achieve statistical significance using indicated statistical tests based on pilot study data. Flow cytometry and Isoplexis cytokine studies utilized 4 mice per experimental group and histological analysis required 3 animals per experimental group unless otherwise indicated.
Data exclusions	One animal was excluded from the data presented in Figure 5C (M2-like macrophage subsets based on intracellular staining of TNF-α and TGF-β). This animal belonged to the day 7 post subcritical injury group. Animal excluded due to sample loss during flow cytometry preparation. All other flow cytometry experiments have n=4 per injury size and timepoint. Sample sizes are indicated in relevant figure caption and methods section.
Replication	In vivo flow cytometry findings are consistent with results from initial pilot studies. Histological quantitative measurements were made from 5 different regions of interest for 3 replicate tissue sections per animal (n=3 animals per experimental group). For second harmonic generation imaging, approximately 15 replicate images within a region of 80µm x 80µm were analyzed for each animal (n=3 per experimental group).
Randomization	Mice were randomly allocated for all in vivo experiments.
Blinding	Researchers were not blinded to allocation during the surgical procedure of performing a subcritical injury vs critical VML injury, as the surgeon was required to use a different biopsy punch. All flow cytometry gating and analysis as well as histological quantifications were performed by a blinded researcher.

Reporting for specific materials, systems and methods

We require information from authors about some types of materials, experimental systems and methods used in many studies. Here, indicate whether each material, system or method listed is relevant to your study. If you are not sure if a list item applies to your research, read the appropriate section before selecting a response.

Materials & experimental systems

n/a	Involved in the study
☐	☒ Antibodies
☒	☐ Eukaryotic cell lines
☒	☐ Palaeontology and archaeology
☐	☒ Animals and other organisms
☒	☐ Clinical data
☒	☐ Dual use research of concern

Methods

n/a	Involved in the study
☒	☐ ChIP-seq
☐	☒ Flow cytometry
☒	☐ MRI-based neuroimaging

Antibodies

Antibodies used	The following antibodies were used in flow cytometry experiments: Zombie viability dye (BioLegend, 1:100 dilution), BV605-conjugated anti-CD4 (BioLegend), BV785-conjugated anti-CD8 (BioLegend), BV421-conjugated anti-CD3 (BioLegend), PerCP-Cy5.5-conjugated anti-CD25 (BioLegend), and APC-conjugated anti-CD127 (BioLegend), BV421, APC-Cy7 or PE-Cy5-conjugated anti-CD11b (BioLegend), APC-Cy7-conjugated anti-Ly6G, BV510 or PerCP-Cy5.5-conjugated anti-Ly6C (BioLegend), BV711 or FITC-conjugated anti-CD64 (BioLegend), PE or APC-conjugated anti-MerTK (BioLegend), PE-Cy7 conjugated anti-CD206 (BioLegend), FITC-conjugated anti-Ly6A/E (BioLegend), APC-conjugated Lineage antibody cocktail (BD Pharmigen), APC-conjugated anti-CD31 (BioLegend), PE-Cy5 conjugated anti-CD29 (BioLegend), and PerCP-Cy5.5-conjugated anti-CXCR4 (BioLegend), BV510-conjugated anti-TNF-α (BioLegend), BV421-conjugated anti-TGF-β (BioLegend), and PE-conjugated anti-SDF-1 (R&D Systems). Antibodies used at 0.25 or 0.5 µg per 100uL staining volume, in accordance with manufacturer recommendation. The following primary antibodies were used for immunostaining: anti-dystrophin (Abcam, ab15277), anti-CD68 (Abcam, ab53444), anti-PDGFRα (Cell Signaling Technology, 3174) and secondary antibodies conjugated to Alexa Fluor 647 (Invitrogen, A21245), Alexa Fluor 555 (Abcam, ab150158), Alexa Fluor 488-conjugated CD206 (BioLegend, 141710), and Alexa Fluor 421 (Abcam, ab175652). Slides mounted with Fluoroshield Mounting Media with DAPI (Abcam, ab104139) where indicated.
Validation	All anti-mouse flow cytometry antibodies have verified reactivity with mouse species and each lot is quality tested for application of flow cytometry. Certificates of analysis are provided from the indicated manufacturer. All primary antibodies used for immunostaining were quality tested for application of immunohistochemistry (IHC) of frozen tissue sections.

Animals and other research organisms

Policy information about studies involving animals; ARRIVE guidelines recommended for reporting animal research, and Sex and Gender in Research

Laboratory animals	C57BL/6J mice were purchased from Jackson Laboratory and maintained as a breeding colony. All animals used in the study were male, 6.1±0.5 (mean ± standard deviation) months in age at the time of euthanasia. Number of mice used for each experiment noted in figure caption and relevant methods section. A total of 60 mice were used to produce the findings presented in this manuscript.
Wild animals	No wild animals were used.
Reporting on sex	Findings in this manuscript apply to male mice.
Field-collected samples	No field-collected samples.
Ethics oversight	All animal studies were approved by the Georgia Institute of Technology Institutional Animal Care and Use Committee.

Note that full information on the approval of the study protocol must also be provided in the manuscript.

Flow Cytometry

Plots

Confirm that:

☒ The axis labels state the marker and fluorochrome used (e.g. CD4-FITC).

☒ The axis scales are clearly visible. Include numbers along axes only for bottom left plot of group (a 'group' is an analysis of identical markers).

☒ All plots are contour plots with outliers or pseudocolor plots.

☒ A numerical value for number of cells or percentage (with statistics) is provided.

Methodology

Sample preparation	Injured (or uninjured for controls) left quadriceps were harvested and digested with 5,500U/ml collagenase II and 2.5U/ml

Sample preparation	Dispase II for 1.5 hours in a shaking 37C water bath. The digested muscles were filtered through a cell strainer to obtain a single cell suspension. Single-cell suspensions were stained for live cells using Zombie NIR (BioLegend) dyes in cell-culture grade PBS per manufacturer instructions. Cells were then fixed in 4% PFA for 10 minutes at 4C. Cells were stained with cell phenotyping antibodies in a 1:1 volume ratio of 3% FBS and Brilliant Stain Buffer (BD Biosciences) according to standard procedures. Samples were protected from light throughout preparation.
Instrument	Cell sorting and flow cytometry data collection was performed on a FACS AriaIII flow cytometer (BD Biosciences).
Software	Flow cytometry data was analyzed with FlowJo software. Following gating of parent cell types, more heterogeneous subsets were visualized and analyzed with UMAP and SPADE (source codes provided in code availability).
Cell population abundance	Purity of cells was ensured by gating only live, single cells before quantification of immune cell phenotypes. All FACS sorting was performed under purity precision mode.
Gating strategy	Cells are first distinguished from debris and doublets using FSC and SSC biplots. Single cells are obtained after sequential FSC-A and FSC-H bi-plot gating. Live cells can be determined using Zombie viability stain. Representative flow plots and gating strategies are included in the supplemental material. Fluorescent minus one (FMO) control samples were used to distinguish negative and positive populations.

☒ Tick this box to confirm that a figure exemplifying the gating strategy is provided in the Supplementary Information.
